# Assembly and comparative analysis of the complete multichromosomal mitochondrial genome of an endangered orchid species, *Calanthe sieboldii*

**DOI:** 10.3389/fpls.2026.1701753

**Published:** 2026-03-10

**Authors:** Tingting Li, Jun Lu, Li Xiong, Guang Zhao, Fang Wu, Jun Yan, Kerui Huang

**Affiliations:** 1 Hunan Provincial Demonstration Center of Forestry Seedling Breeding, Changsha, China; 2 Hunan Edible Fungal Research Institute, Changsha, China; 3 College of Life and Environmental Sciences, Hunan University of Arts and Science, Changde, China

**Keywords:** *Calanthe sieboldii*, mitogenomes, orchidaceae, phylogenetic analysis, RNA editing

## Abstract

**Introduction:**

Plant mitochondrial genomes (mitogenomes) are known for their structural complexity, particularly within the Orchidaceae. To understand the evolutionary dynamics in the endangered genus *Calanthe*, this study provides the first complete mitogenome assembly for the endangered species *Calanthe sieboldii*, a species of horticultural and conservation importance.

**Methods:**

A hybrid sequencing approach combining Nanopore long reads and BGI short reads was used for denovo assembly. The genome was annotated, and we performed comparative analyses of repetitive sequences, interorganellar DNA transfer, codon usage, RNA editing, synteny, and phylogeny.

**Results:**

The 644,236 bp mitogenome exhibits a highly fragmented architecture, comprising 21 independent circular chromosomes ranging from 19.9 to 48.7 kb. We annotated 39 unique protein-coding genes, 23 tRNA genes, and 3 rRNA genes. The genome is characterized by a high density of repetitive sequences and a massive influx of chloroplast DNA, with mitochondrial–plastid sequences accounting for 12.72% of the total length. Comparative synteny analysis with other orchid species revealed an almost complete loss of gene order, highlighting extreme structural rearrangement. Despite this plasticity, core molecular features, such as codon usage and predicted RNA editing patterns, remain conserved. Phylogenetic analysis robustly placed *C. sieboldii* within the Orchidaceae.

**Discussion:**

This study decodes a complex multichromosomal mitogenome, reinforcing the paradigm of dynamic structural evolution in orchids and providing a vital genomic resource to support conservation efforts and evolutionary research on the *Calanthe* genus.

## 1 Introduction

Plant mitochondrial genomes (PMGs) are renowned for their remarkable evolutionary dynamics, often described as an “evolutionary paradox” where slow rates of nucleotide substitution coincide with rapid and extensive structural rearrangement (Fan et al., 2022; Wang et al., 2023a). Unlike the compact and conserved mitogenomes of animals, PMGs are characterized by their large and highly variable size, low gene density, and complex architectures. These genomes frequently deviate from the simple, canonical circular model (Møller et al., 2021). This structural plasticity is largely driven by a high frequency of homologous recombination, which is fueled by an abundance of repetitive sequences, including tandem, palindromic, and other dispersed repeats (Zhong et al., 2022; Ni et al., 2023; Jiang et al., 2023). Furthermore, PMGs are known to act as “melting pots” for foreign DNA, readily incorporating large segments from the chloroplast genome (mitochondrial–plastid sequences; MTPTs) and the nuclear genome. These foreign insertions contribute importantly to their size and structural complexity (Anderson et al., 2021; Zhou S. X. et al., 2023; Han et al., 2024). To ensure the proper expression of their conserved gene repertoire, which is essential for cellular respiration and biogenesis, PMGs rely on extensive post-transcriptional modifications, most notably C-to-U RNA editing, a process that can correct codons and is vital for producing functional proteins (Small et al., 2023; Wang et al., 2023). The advent of long-read sequencing and graph-based assembly methods has been instrumental in moving beyond the simplistic “master circle” model, revealing that many PMGs exist as complex, dynamic populations of linear, branched, and multicircular molecules (Fischer et al., 2022; He et al., 2023; Lee et al., 2023; Bi et al., 2024).

This pattern of extreme structural dynamism is particularly pronounced in the Orchidaceae, which is among the most rapidly diversifying and species-rich lineages of flowering plants (Jacquemyn et al., 2023; Zhang et al., 2023). Recent studies utilizing long-read sequencing have consistently revealed that the mitogenomes of many orchid species are not single molecules but are highly fragmented into multiple, independent circular chromosomes. For instance, complex multicircular architectures have been documented in diverse orchid genera, including *Dendrobium* (20–25 chromosomes) (Wang et al., 2023b; Wang et al., 2024), *Cymbidium* (19 chromosomes) (Shen et al., 2024), *Paphiopedilum* (26 chromosomes) (Yang et al., 2023), and the mycoheterotrophic genus *Epipogium* (26 chromosomes) (Zhao et al., 2024). These findings suggest that frequent genomic fragmentation and rearrangement, often accompanied by massive influxes of chloroplast DNA, are hallmarks of mitogenomic evolution within this family (Wang et al., 2023b; Yang et al., 2023). This high degree of genomic instability provides a potential molecular basis for the rapid speciation and adaptive radiation that characterize the Orchidaceae.

The genus *Calanthe*, comprising more than 200 species, is a large and taxonomically complex group within the Orchidaceae that is well known for its significant horticultural value and use in traditional medicine (Chung et al., 2013; Kim et al., 2021; Pengdee et al., 2025). Despite the availability of sequences for a growing number of orchid mitogenomes, a complete, high-resolution characterization of the mitochondrial genome (mitogenome) for a representative species of *Calanthe* has not been reported to date. This represents a notable knowledge gap, limiting our understanding of the evolutionary dynamics within this important orchid lineage. Therefore, the primary objectives of this study were to (1) utilize long-read sequencing to assemble and annotate the complete mitogenome of *C. sieboldii*, (2) comprehensively characterize its mitogenomic architecture and critical molecular features, including gene content, repeat landscape, codon usage bias, RNA editing patterns, and the extent of interorganellar DNA transfer, and (3) place these features into a broader evolutionary context by conducting comparative synteny and phylogenetic analyses. By resolving the intricate structure of the *C. sieboldii* mitogenome, this research provides a foundational genomic resource and offers critical insights into the extreme structural plasticity that defines mitogenome evolution in the Orchidaceae.

## 2 Materials and methods

### 2.1 Plant material, DNA extraction, and sequencing

Fresh, young leaves of *Calanthe sieboldii* were collected from Xishan State-owned Forest Farm, Linwu County, Hunan Province, China. The leaf tissues were immediately flash-frozen in liquid nitrogen and stored at −80 °C until DNA extraction. Total genomic DNA was isolated from approximately 100 mg of leaf tissue using a modified cetyltrimethylammonium bromide method. The quality and quantity of extracted DNA were assessed using a NanoDrop 2000 spectrophotometer (Thermo Fisher Scientific, USA) and 1.0% agarose gel electrophoresis.

A hybrid sequencing strategy was employed to generate long and short reads. High-coverage long-read sequencing was performed on the Oxford Nanopore Technologies PromethION platform. Concurrently, high-accuracy short-read sequencing was conducted using the BGI-seq platform.

### 2.2 Mitochondrial genome assembly and annotation

*De novo* assembly of the *C. sieboldii* mitogenome was performed in a multistep process. First, an initial draft assembly was generated from the Nanopore long reads using Flye v2.9.2-b1786 (Kolmogorov et al., 2019) with default parameters. The resulting assembly graph was visualized with Bandage v0.8.1 (Wick et al., 2015) to assess its complexity. To identify mitochondrial contigs, a local BLAST database was created from all assembled contigs. These contigs were then queried using the complete mitochondrial gene sets from six related orchid species (*Dendrobium nobile*, OR413867.1–OR413891.1; *Gastrodia elata*, PP239863.1–PP239927.1; *Phalaenopsis mannii*, PQ180326.1–PQ180344.1; *Cymbidium ensifolium*, OR754263.1–OR754281.1; *Danxiaorchis yangii*, PQ306624.1; and *Bletilla striata*, PQ859845.1–PQ859864.1) via BLASTn with the following parameters: -evalue 1e-5 -outfmt 6 -max_hsps 10 -word_size 7 -task blastn-short.

Next, both the Nanopore long reads and BGI short reads were mapped to the identified mitochondrial contigs using minimap2 v2.26-r1175 (Li, 2018). The mapped reads were filtered and extracted for final hybrid assembly. This was performed using Unicycler v0.5.0 (Wick et al., 2017) in its hybrid mode with default settings. The final, polished assembly graph was visualized again with Bandage v0.8.1 (Wick et al., 2015) to confirm the complete, multipartite structure of the mitogenome.

We performed a two-step long-read validation followed by alignment-based visualization. First, Nanopore reads were mapped to each assembled mitochondrial molecule using minimap2 (Li, 2018), and the successfully aligned reads were extracted as supporting evidence for downstream analyses. Next, these mitochondria-supported reads were aligned back to each of the 21 circular-mapping contigs using nucmer algorithms in the software package MUMmer (https://github.com/mummer4/mummer), and the alignments were visualized to inspect read continuity at the putative circularization junctions (**Supplementary Figures 1**-**S21**). Across all 21 molecules, we consistently observed long reads spanning the breakpoint, with continuous alignments bridging the contig end and start regions (i.e., end-to-start support). This robust junction-spanning read evidence demonstrates that each contig represents a genuine circular-mapping mitochondrial molecule and confirms that both our assembly and the inferred ring-splitting (multipartite) structure are reliable rather than artifacts.

Gene annotation was conducted using a combination of automated tools and manual curation. Protein-coding genes (PCGs) were initially annotated using the Plant Mitochondrial Genome Annotator (PMGA) web server (Li et al., 2024), with the aforementioned orchid species as references. Transfer RNA (tRNA) genes were identified using tRNAscan-SE v2.0.11 (Lowe and Eddy, 1997) with default parameters for organellar genomes. Ribosomal RNA (rRNA) genes were located by BLASTn searches against the reference rRNA sequences. All annotations were manually verified and corrected for start/stop codons and intron/exon boundaries using Apollo v1.11.8 (Lewis et al., 2002). The final circular maps of the mitochondrial chromosomes were generated using the OrganellarGenomeDRAW (OGDRAW) tool (Stephan et al., 2019).

### 2.3 Analysis of repetitive sequences

Three categories of repetitive sequences were identified in the *C. sieboldii* mitogenome. Simple sequence repeats were detected using MISA v2.1 (Beier et al., 2017). Tandem repeats were identified using Tandem Repeats Finder (TRF) v4.09 (Benson, 1999). Dispersed repeats, including forward (direct), palindromic (inverted), reverse, and complement repeats, were identified using the REPuter web server (Stefan et al., 2001) with a minimum repeat size of 30 bp and sequence identity ≥ 90% (Hamming distance = 3). The distribution of these repeats was visualized using custom scripts and the Circos package v0.69.9 in R (Zhang et al., 2013).

### 2.4 Codon usage and RNA editing analysis

The coding sequences of all unique PCGs were extracted using PhyloSuite v1.1.16 (Zhang et al., 2020). The relative synonymous codon usage (RSCU) values and overall codon usage patterns were calculated for all PCGs using MEGA v7.0 (Kumar et al., 2016).

As transcriptomic data were not available, C-to-U RNA editing sites were predicted *in silico*. The complete set of PCGs was analyzed using Deepred-mt (Edera et al., 2021), a deep learning-based tool optimized for prediction of RNA editing in plant mitochondria. Only editing sites with prediction probability > 0.9 were retained and reported.

### 2.5 Interorganellar DNA transfer analysis

To identify sequences transferred from the chloroplast genome (MTPTs), the complete chloroplast genome of *C. sieboldii* was first assembled from the same sequencing data using GetOrganelle v1.7.7.1 (Jin et al., 2020) and annotated with CPGAVAS2 (Shi et al., 2019). The complete mitochondrial and chloroplast genomes were then aligned using BLASTn v2.13.0 (Chen et al., 2015) to identify regions of high homology. The results were visualized as a circular diagram using the R package RCircos (Zhang et al., 2013).

### 2.6 Comparative synteny analysis

To assess the structural evolution of the *C. sieboldii* mitogenome, whole-genome synteny analysis was conducted against four other Orchidaceae species: *Danxiaorchis yangii*, *Dendrobium nobile*, *Cymbidium ensifolium*, and *Phalaenopsis mannii*. Homologous blocks between genomes were identified using BLASTn with the following parameters: -evalue 1e-5 -word_size 9 -gapopen 5 -gapextend 2 -reward 2 -penalty -3. The resulting collinear blocks were filtered to retain only those longer than 500 bp and visualized using the Multiple Synteny Plot function within MCscanX (Wang et al., 2012).

### 2.7 Phylogenetic analysis

To evaluate the phylogenetic placement of *C. sieboldii*, based on mitogenome sequences, a dataset of 24 conserved mitochondrial PCGs (*atp1*, *atp4*, *atp8*, *atp9*, *ccmB*, *ccmC*, *ccmFC*, *ccmFN*, *cob*, *cox1*, *cox2*, *cox3*, *matR*, *mttB*, *nad1*, *nad2*, *nad3*, *nad4*, *nad4L*, *nad5*, *nad6*, *nad7*, *nad9*, and *rps12*) from 33 angiosperm species was compiled, with two species from the Alismatales serving as the outgroup. The homologous gene sequences were extracted using PhyloSuite v1.1.16 (Zhang et al., 2020) and aligned using MAFFT v7.505 (Katoh and Standley, 2013). The alignments were then concatenated into a supermatrix.

Phylogenetic reconstruction was performed using the maximum likelihood (ML) and Bayesian inference (BI) methods. The ML tree was constructed using IQ-TREE v1.6.12 (Nguyen et al., 2015), with the best-fit substitution model automatically selected by ModelFinder. Branch support was assessed using 1,000 bootstrap (BS) replicates (ultrafast bootstrap and SH-aLRT). The BI analysis was performed using MrBayes v3.2.7 (Ronquist and Huelsenbeck, 2003), with the GTR+I+G model selected. Two independent runs of four Markov chain Monte Carlo chains were executed for 2,000,000 generations, with trees sampled every 1,000 generations. The initial 25% of trees were discarded as burn-in, and convergence was confirmed when the average standard deviation of split frequencies decreased below 0.01. The resulting phylogenetic trees were visualized and annotated using the Interactive Tree Of Life (iTOL) v6 (Letunic and Bork, 2019).

## 3 Results

### 3.1 Mitogenome assembly reveals a complex, multicircular architecture

**Figure 1:**
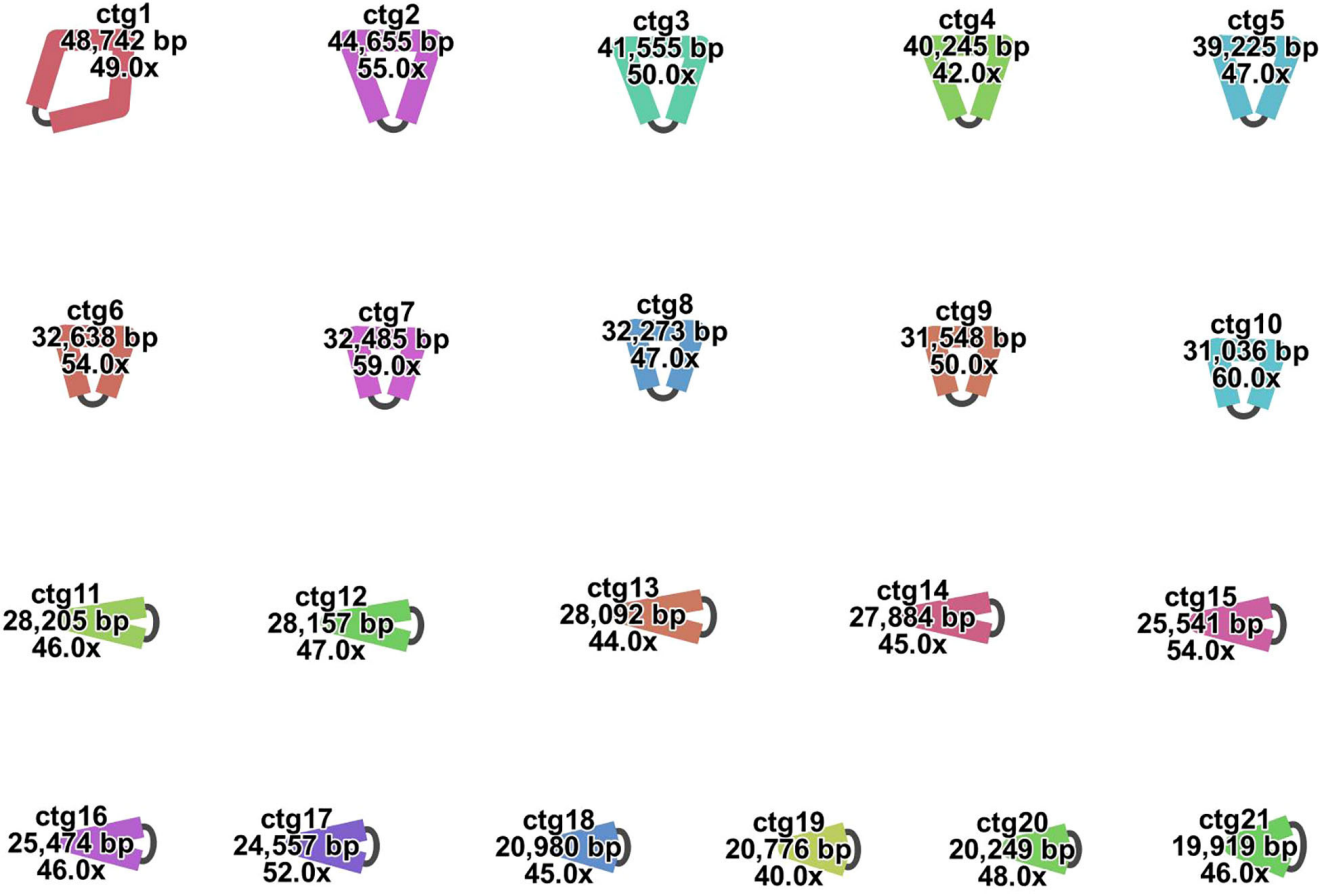
Multipartite architecture of the *Calanthe sieboldii* mitochondrial genome.

The complete mitogenome of *Calanthe sieboldii* was sequenced using long-read technology and assembled into a complex, multipartite structure. Visualization of the draft assembly graph using Bandage software revealed a primarily branched morphology, which resolved into 21 distinct, self-circularized contigs, hereafter referred to as chromosomes (**Figure 1**). This multichromosomal architecture is consistent with the highly fragmented mitogenomes recently characterized in other advanced orchid genera, such as *Dendrobium* (Wang et al., 2023b; Wang et al., 2024), *Cymbidium* (Shen et al., 2024), and *Paphiopedilum* (Yang et al., 2023), but stands in stark contrast to the single-circle or simpler isomeric structures found in many other angiosperms, including the related monocot *Asparagus officinalis* (Sheng et al., 2023).

The 21 individual circular chromosomes (ctg1 to ctg21) constitute the complete mitogenome, as resolved from the final assembly graph and visualized using Bandage software. Each contig is shown as an independent, self-circularized molecule. The labels provide the contig name, its total length in base pairs (bp), and the average sequencing coverage (x). This complex structure highlights the highly fragmented and multichromosomal structure of the *C. sieboldii* mitogenome.

The total assembled length of the *C. sieboldii* mitogenome is 644,236 bp, with an overall GC content of 43.53%. The 21 circular chromosomes exhibit significant heterogeneity in size and sequence composition (**Table 1**). The chromosome lengths range from 19,919 bp for Chromosome (Chr) 21 to 48,742 bp for Chr1, and their individual GC contents vary between 38.26% and 45.86%. This structural and compositional diversity underscores the highly dynamic nature of the orchid mitogenome.

**Table 1:** Characteristics of the assembled multipartite mitochondrial genome of *Calanthe sieboldii*.

GenBank accession	Molecule	Topology	Length (bp)	GC content (%)
PX168812–PX168832	Total	–	644,236	43.53
PX168822	Chromosome 1	Circular	48,742	45.36
PX168825	Chromosome 2	Circular	44,655	44.21
PX168826	Chromosome 3	Circular	41,555	45.31
PX168827	Chromosome 4	Circular	40,245	45.37
PX168828	Chromosome 5	Circular	39,225	42.39
PX168829	Chromosome 6	Circular	32,638	42.84
PX168830	Chromosome 7	Circular	32,485	40.89
PX168831	Chromosome 8	Circular	32,273	43.32
PX168832	Chromosome 9	Circular	31,548	43.93
PX168812	Chromosome 10	Circular	31,036	43.77
PX168813	Chromosome 11	Circular	28,205	45.86
PX168814	Chromosome 12	Circular	28,157	44.42
PX168815	Chromosome 13	Circular	28,092	42.09
PX168816	Chromosome 14	Circular	27,884	40.63
PX168817	Chromosome 15	Circular	25,541	44.43
PX168818	Chromosome 16	Circular	25,474	44.2
PX168819	Chromosome 17	Circular	24,557	44.96
PX168820	Chromosome 18	Circular	20,980	42.65
PX168821	Chromosome 19	Circular	20,776	38.26
PX168823	Chromosome 20	Circular	20,249	43.11
PX168824	Chromosome 21	Circular	19,919	41.79

### 3.2 Gene content and annotation of the mitogenome

**Figure 2:**
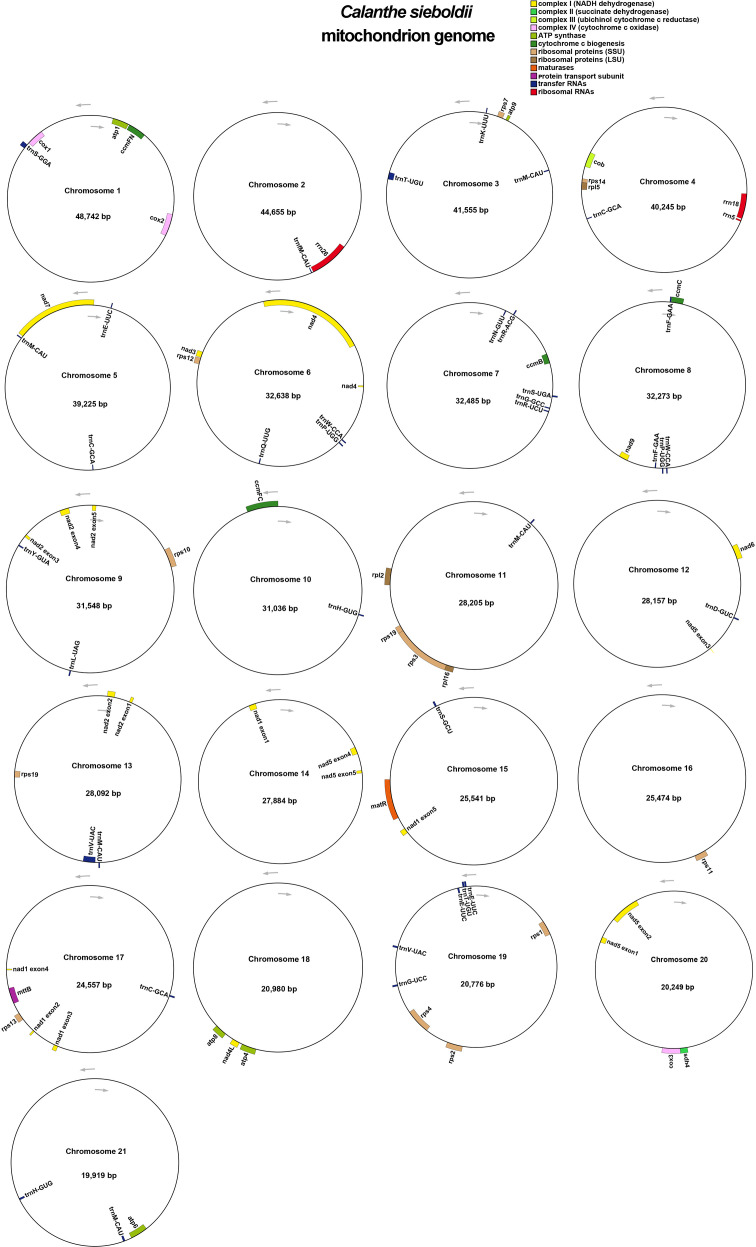
Gene maps of the 21 mitochondrial chromosomes of *Calanthe sieboldii*. The annotation and organization of genes across the 21 circular mitochondrial chromosomes are shown. Genes are color-coded based on their functional categories, as indicated in the key at the top right.

Annotation of the *C. sieboldii* mitogenome identified a total of 65 unique genes, which comprise 39 PCGs, 23 tRNA genes, and 3 rRNA genes (**Figure 2**, **Table 2**). Among the PCGs, the gene encoding ribosomal small subunit protein 19 (*rps19*) was present in two copies, bringing the total number of PCGs to 40. This gene complement is largely consistent with that reported for other orchids, such as *Dendrobium* (Wang et al., 2023a), although specific gene losses and gains are known to occur frequently within the Orchidaceae.

**Table 2:** Genes annotated in the mitochondrial genome of *Calanthe sieboldii*.

Gene group	Gene names
ATP synthase	*atp*1, *atp*4, *atp*6, *atp*8, *atp*9
NADH dehydrogenase	*nad*1, *nad*2, *nad*3, *nad*4, *nad*4L, *nad*5, *nad*6, *nad*7, *nad*9
Cytochrome *b*	*cob*
Cytochrome *c* biogenesis	*ccm*B, *ccm*C, *ccm*FC, *ccm*FN
Cytochrome *c* oxidase	*cox*1, *cox*2, *cox*3
Maturases	*mat*R
Protein transport subunit	*mtt*B
Ribosomal protein large subunit	*rpl*2, *rpl*5, *rpl*16
Ribosomal protein small subunit	*rps*1, *rps*2, *rps*3, *rps*4, *rps*7, *rps*10, *rps*11, *rps*12, *rps*13, *rps*14, *rps*19(×2)
Succinate dehydrogenase	*sdh*4
Ribosome RNA	*rrn*5, *rrn*18, *rrn*26
Transfer RNA	*trn*C-GCA(×3), *trn*D-GUC, *trn*E-UUC(×3), *trn*F-GAA(×2), *trnf*M-CAU, *trn*G-GCC, *trn*G-UCC, *trn*H-GUG(×2), *trn*K-UUU, *trn*L-UAG, *trn*M-CAU(×5), *trn*N-GUU(×2), *trn*P-UGG(×2), *trn*Q-UUG, *trn*R-ACG, *trn*R-UCU, *trn*S-GCU, *trn*S-GGA, *trn*S-UGA, *trn*T-UGU(×2), *trn*V-UAC(×2), *trn*W-CCA(×2), *trn*Y-GUA

The 39 unique PCGs were further classified into 24 core genes and 15 non-core genes (**Figure 2**, **Table 2**). The core gene set, which is highly conserved across land plants, comprises five ATP synthase subunits (*atp1*, *atp4*, *atp6*, *atp8*, and *atp9*), nine NADH dehydrogenase subunits (*nad1*, *nad2*, *nad3*, *nad4*, *nad4L*, *nad5*, *nad6*, *nad7*, and *nad9*), three cytochrome *c* oxidase subunits (*cox1*, *cox2*, and *cox3*), four cytochrome *c* biogenesis factors (*ccmB*, *ccmC*, *ccmFC*, and *ccmFN*), and one gene each for cytochrome *b* (*cob*), maturase (*matR*), and a transport membrane protein (*mttB*). The 15 non-core genes, which are more evolutionarily labile, comprise 11 ribosomal small subunit genes (*rps1*, *rps2*, *rps3*, *rps4*, *rps7*, *rps10*, *rps11*, *rps12*, *rps13*, *rps14*, and *rps19*), three ribosomal large subunit genes (*rpl2*, *rpl5*, and *rpl16*), and one succinate dehydrogenase subunit gene (*sdh4*).

Annotation of RNA genes revealed a complete set of rRNAs (**Figure 2**, **Table 2**), comprising *rrn5*, *rrn18*, and *rrn26*. Furthermore, 23 unique tRNA genes capable of encoding all 20 standard amino acids were identified. A notable feature of the *C. sieboldii* mitogenome is the high frequency of tRNA gene duplication. Ten of the 23 unique tRNA genes were present in multiple copies, resulting in a total of 38 tRNA genes. The gene *trnM-CAU* was the most abundant with five copies, while *trnC-GCA* and *trnE-UUC* each consisted of three copies. Seven other tRNA genes (*trnF-GAA*, *trnH-GUG*, *trnN-GUU*, *trnP-UGG*, *trnT-UGU*, *trnV-UAC*, and *trnW-CCA*) were present in two copies each, ensuring a robust translational machinery within the mitochondrion.

### 3.3 The landscape of repetitive sequences and interorganellar DNA transfer

**Figure 3:**
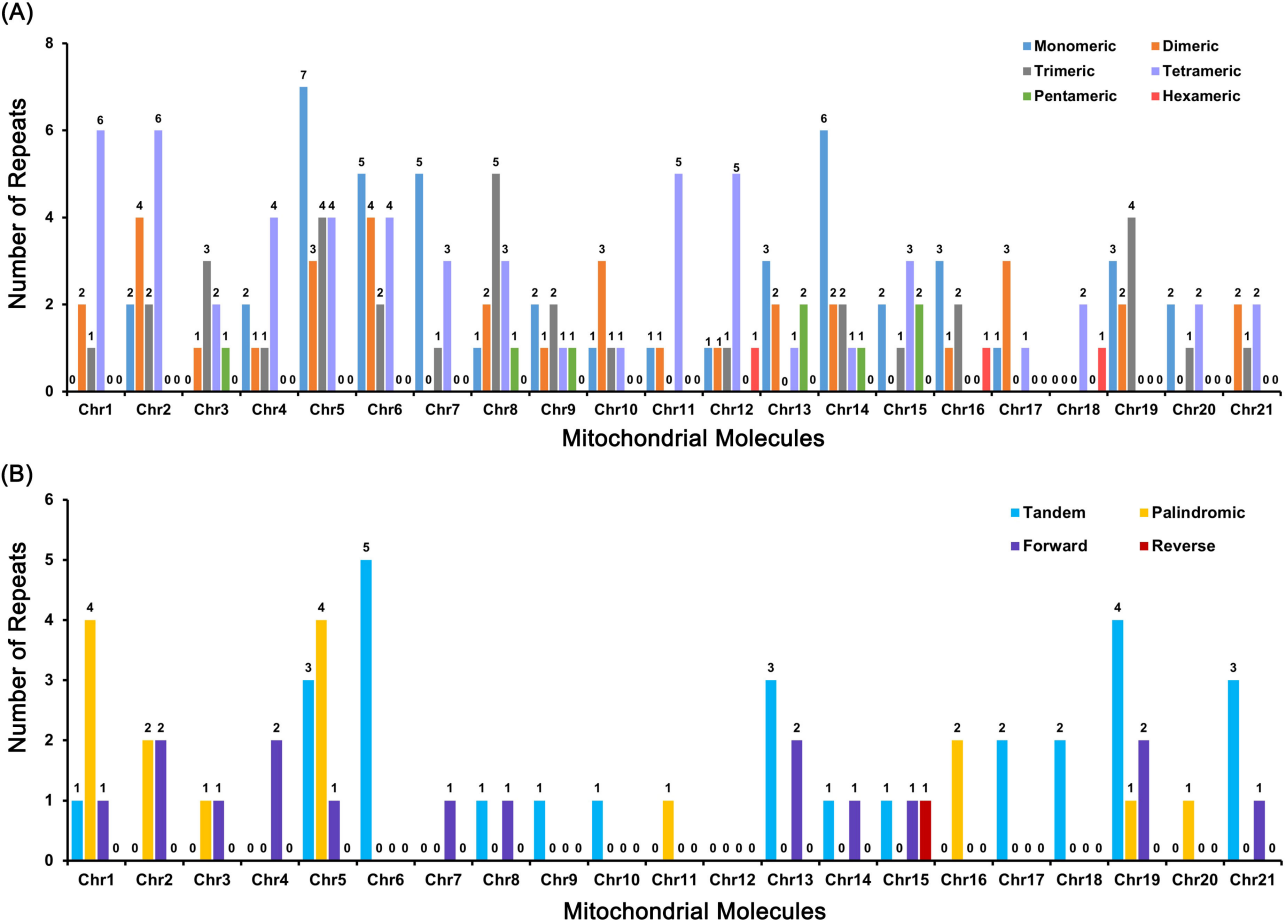
Analysis of repetitive sequences across the 21 mitochondrial chromosomes of *Calanthe sieboldii*. The number and types of repetitive sequences on each chromosome (Chr) is shown. **(A)** Distribution of simple sequence repeats, categorized by the length of the repeat unit from monomeric (mononucleotide) to hexameric (hexanucleotide). **(B)** Distribution of dispersed repeats, classified as tandem, palindromic (inverted), forward (direct), and reverse. The *y*-axis indicates the total number of each specific repeat type identified on each chromosome, highlighting their abundance and uneven distribution.

To investigate the drivers of the observed structural complexity, we analyzed the repetitive DNA content of the *C. sieboldii* mitogenome. The genome is rich in simple and dispersed repeats, which are unevenly distributed across the 21 chromosomes (**Figure 3**). Analysis of simple sequence repeats revealed that mononucleotide and tetranucleotide repeats were the most abundant types (**Figure 3A**). Mononucleotide repeats were most frequent on Chr5 (seven instances) and Chr14 (six instances), whereas tetranucleotide repeats were most numerous on Chr1 and Chr2 (six instances each). Dispersed repeat analysis showed that tandem and palindromic repeats were the predominant forms (**Figure 3B**). Tandem repeats were most concentrated on Chr6 (five instances) and Chr19 (four instances), whereas palindromic repeats were most abundant on Chr1 and Chr5 (four instances each). The abundance of these repeats, particularly tandem and palindromic structures, provides a rich substrate for the frequent homologous recombination events known to drive genomic rearrangement and fragmentation in plant mitogenomes (Zhong et al., 2022; Ni et al., 2023; Jiang et al., 2023).

**Figure 4:**
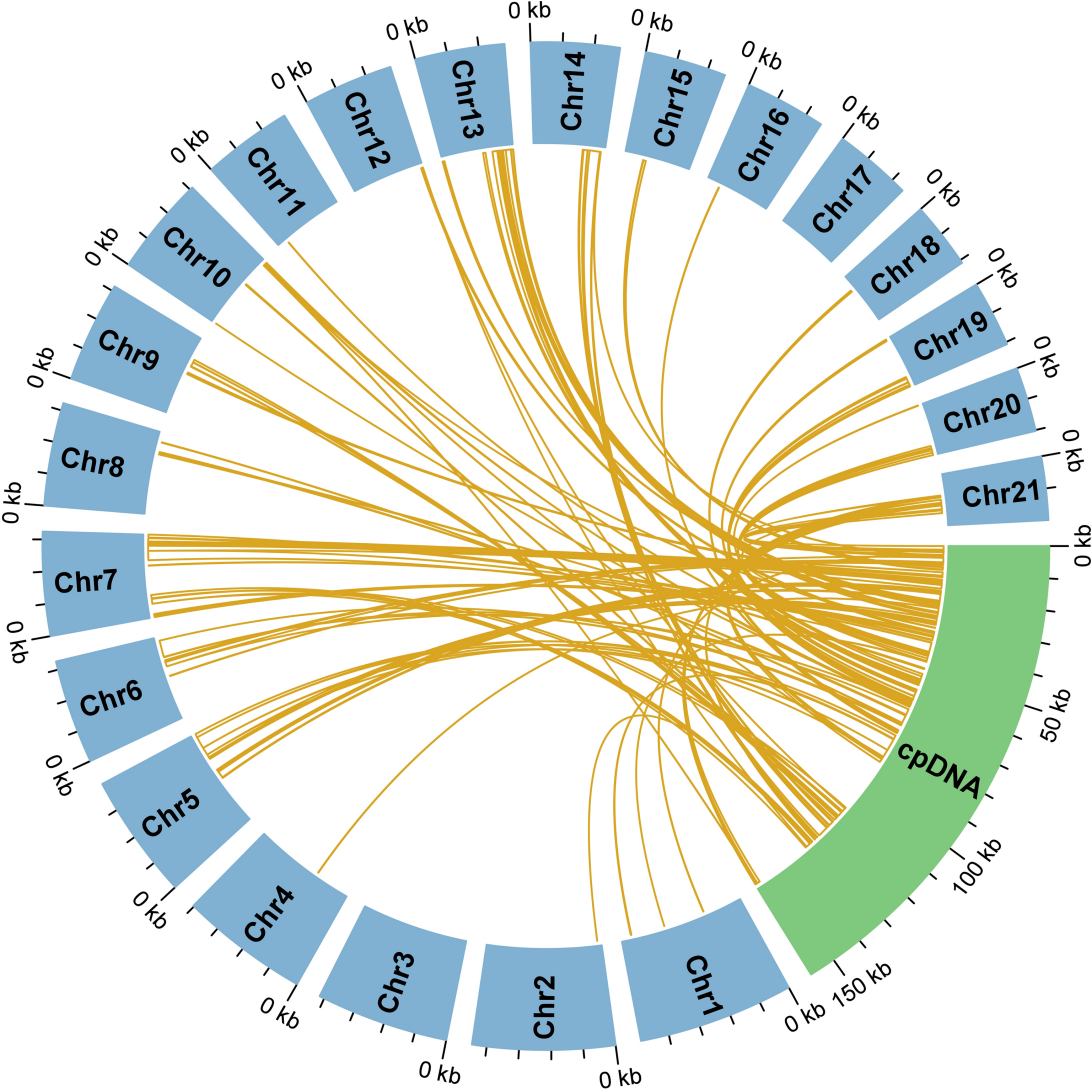
Sequence homology between the mitochondrial and chloroplast genomes of *Calanthe sieboldii*. The circular diagram visualizes the extent of interorganellar DNA transfer. The 21 mitochondrial chromosomes (Chr1–Chr21) are represented by the blue blocks, and the complete chloroplast genome (cpDNA) is represented by the green block. The gold lines connect regions of high sequence homology, illustrating the distribution of the 96 identified mitochondrial–plastid sequences that have been transferred from the chloroplast genome to mitochondrial chromosomes.

In addition to endogenous repeats, the *C. sieboldii* mitogenome is characterized by a massive influx of DNA from the chloroplast genome. We identified 96 independent MTPTs with a cumulative length of 81,957 bp, accounting for an exceptionally high 12.72% of the total mitogenome length (**Figure 4**). This proportion is substantially greater than that reported for many other angiosperms, such as *Prunus salicina* (0.6%) (Fang et al., 2021) and *Ilex metabaptista* (2.10%) (Zhou et al., 2023), but is remarkably consistent with the high degree of MTPT integration observed in other dynamically evolving orchid mitogenomes, including *Dendrobium* (10.5%–12.0%) (Wang et al., 2023a) and *Paphiopedilum* (10.34%) (Yang et al., 2023).

Annotation of these MTPTs revealed that they are not merely non-functional fragments but carry a large complement of intact, chloroplast-derived genes (**Supplementary Table 1**). A total of 47 intact genes were identified within these transferred regions, including 34 PCGs and 13 tRNA genes. The transferred PCGs span a broad range of functions and include *accD*, *atpI*, *infA*, *ndhA*, *ndhE*, *ndhI*, *petD*, *petG*, *petL*, *petN*, *psaC*, *psaI*, *psaJ*, *psbA*, *psbE*, *psbF*, *psbH*, *psbL*, *psbM*, *psbZ*, *rpl14*, *rpl16*, *rpl20*, *rpl32*, *rpl33*, *rpl36*, *rpoA*, *rps11*, *rps16*, *rps18*, *rps19*, *rps2*, *rps3*, and *rps8*. The presence of these intact genes, together with 13 functionally complete tRNA genes, provides strong evidence for large-scale gene transfer between the chloroplast and mitochondrion genomes during the evolution of *C. sieboldii*.

### 3.4 Codon usage bias and RNA editing features

**Figure 5:**
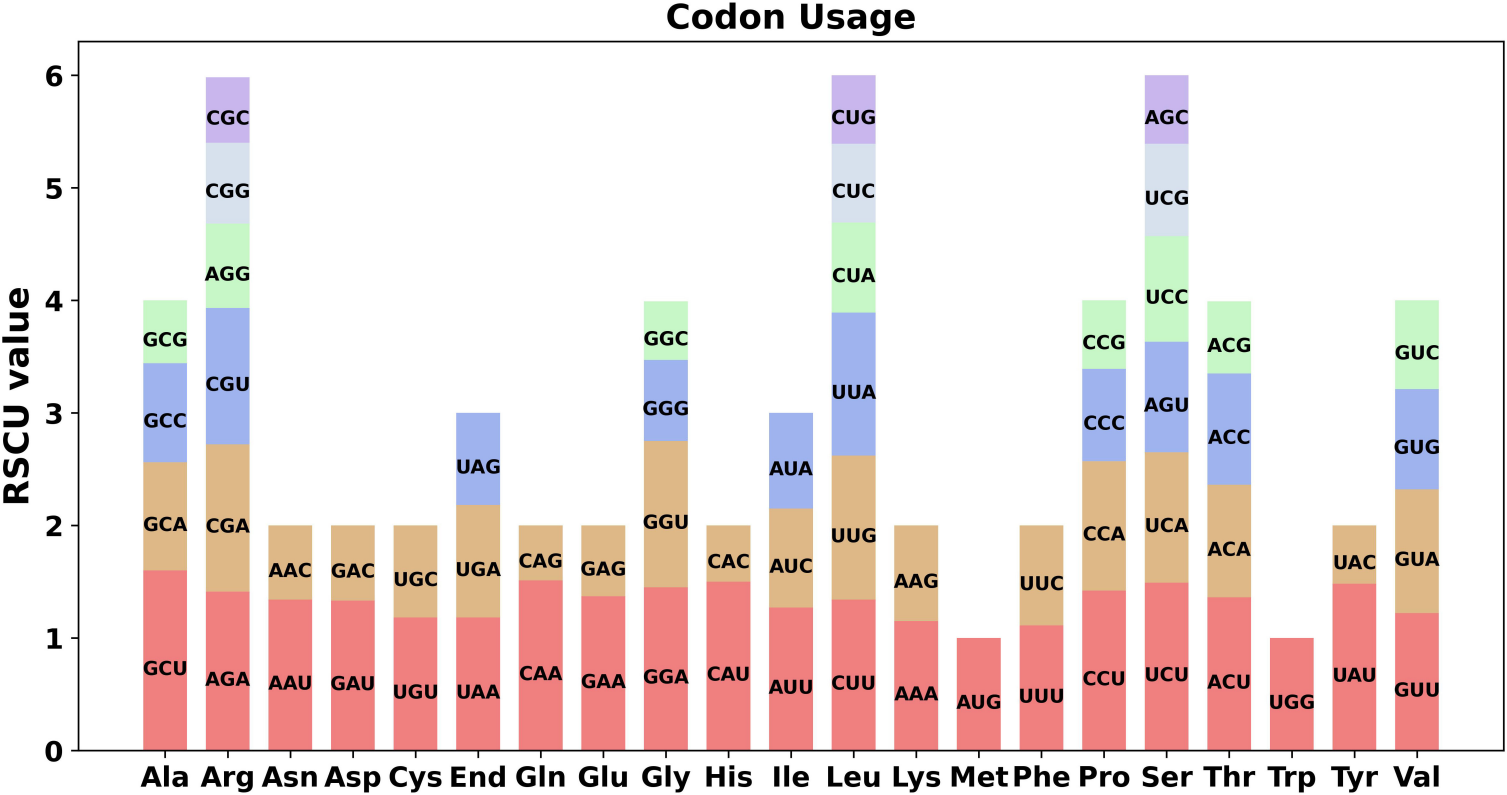
Relative synonymous codon usage (RSCU) among *Calanthe sieboldii* mitochondrial protein-coding genes. The stacked bar chart shows the codon usage bias for the 39 unique protein-coding genes in the mitogenome. The *x*-axis represents the amino acids and the stop codon (End). The *y*-axis indicates the RSCU value; codons with RSCU > 1.0 are more frequent. The chart illustrates a strong preference for codons ending in A or U, which is a common feature in plant mitogenomes.

Analysis of the 39 unique PCGs revealed a significant codon usage bias in the *C. sieboldii* mitogenome. The RSCU values (**Figure 5**) revealed a strong preference for codons ending in A or U. Of the 30 preferred codons with RSCU values greater than 1.0, 24 (80%) had A or U at the third position. This bias was particularly evident in two-fold degenerate codons; for example, the usage of CAU for histidine (RSCU = 1.50) was three times that of CAC (RSCU = 0.50), and the usage of CAA for glutamine (RSCU = 1.51) was much higher than its synonymous counterpart. This A/U preference is a common feature of plant mitogenomes and has been reported in diverse lineages (He et al., 2023; Tang et al., 2023). The optimal codons for the four-fold degenerate alanine and the six-fold degenerate leucine were GCU (RSCU = 1.60) and CUU (RSCU = 1.34), respectively. The stop codon UAA (RSCU = 1.18) was the most frequently used.

**Figure 6:**
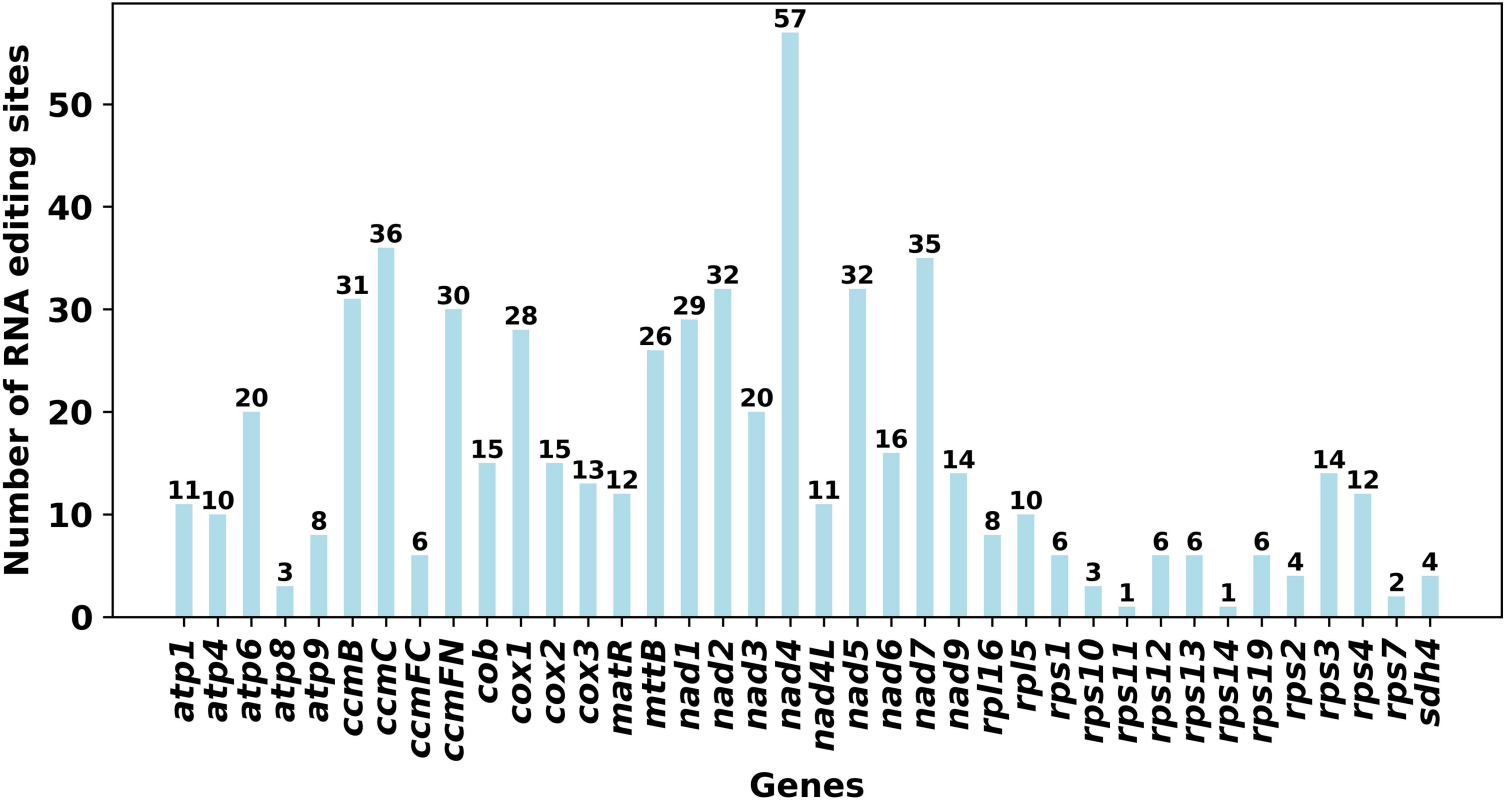
Predicted C-to-U RNA editing sites in the protein-coding genes of the *Calanthe sieboldii* mitogenome. The bar chart displays the number of predicted C-to-U RNA editing sites for each of the 39 unique protein-coding genes. The *x-*axis lists the individual genes, and the *y*-axis indicates the total count of editing sites per gene.

We predicted a total of 574 C-to-U RNA editing sites across the 39 unique PCGs, highlighting the critical role of this post-transcriptional modification in maintaining mitochondrial function (**Figure 6**). While the absolute number of sites remains to be validated transcriptomically, the distribution of these predicted sites revealed distinct patterns across gene families. The number of putative editing sites varied dramatically among genes. The NADH dehydrogenase subunit 4L gene (*nad4L*) was the most frequently edited, containing 57 putative sites. Other genes predicted to contain a high density with a high frequency of editing sites included *ccmC* (36), *nad7* (35), *nad2* (32), *nad5* (32), and *ccmB* (31). In contrast, *rps11* and *rps14* each had only a single editing site. This differential editing pattern, with hotspots concentrated in the NADH dehydrogenase (*nad*) and cytochrome *c* biogenesis (*ccm*) gene families and very few sites in ribosomal protein (*rps*) genes, is highly consistent with patterns observed in other angiosperm mitogenomes (Wang et al., 2023; Han et al., 2024), suggesting a conserved regulatory mechanism that prioritizes the functional integrity of core bioenergetic protein complexes.

### 3.5 Comparative synteny and phylogenetic analyses

**Figure 7:**
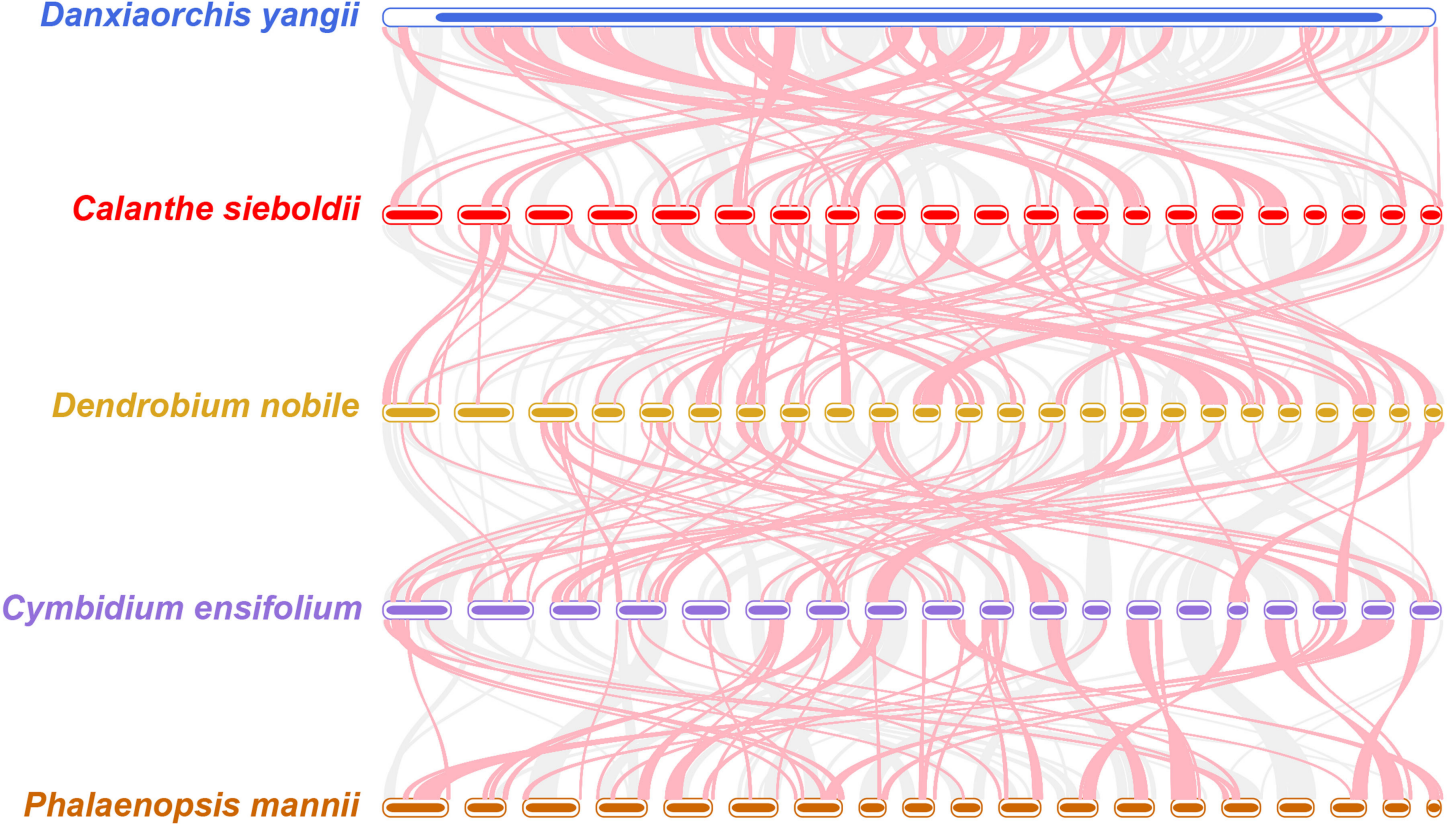
Comparative synteny analysis of the *Calanthe sieboldii* mitogenome with other Orchidaceae species. The figure presents a whole-genome synteny comparison between the mitogenome of *Calanthe sieboldii* and those of four other representative orchid species: *Danxiaorchis yangii*, *Dendrobium nobile*, *Cymbidium ensifolium*, and *Phalaenopsis mannii*. Each horizontal track represents the concatenated mitochondrial chromosomes (or contigs) for a single species. The ribbons connecting the tracks represent homologous syntenic blocks.

To investigate the structural evolution of the *C. sieboldii* mitogenome, we performed a comparative synteny analysis against the mitogenomes of four other Orchidaceae species: *Danxiaorchis yangii*, *Dendrobium nobile*, *Cymbidium ensifolium*, and *Phalaenopsis mannii* (**Figure 7**). The analysis revealed an extremely low degree of collinearity, characterized by highly fragmented and crossed syntenic blocks with almost no long, conserved regions. This pattern, particularly evident when comparing the 21 chromosomes of *C. sieboldii* to the mitogenome of *D. yangii*, is consistent with findings in other studies of orchids that report a near-complete loss of gene-order conservation (Yang et al., 2023; Shen et al., 2024). These results provide strong genomic evidence for the highly dynamic evolutionary nature of Orchidaceae mitogenomes, which have undergone frequent events of fragmentation, recombination, and inversion.

**Figure 8:**
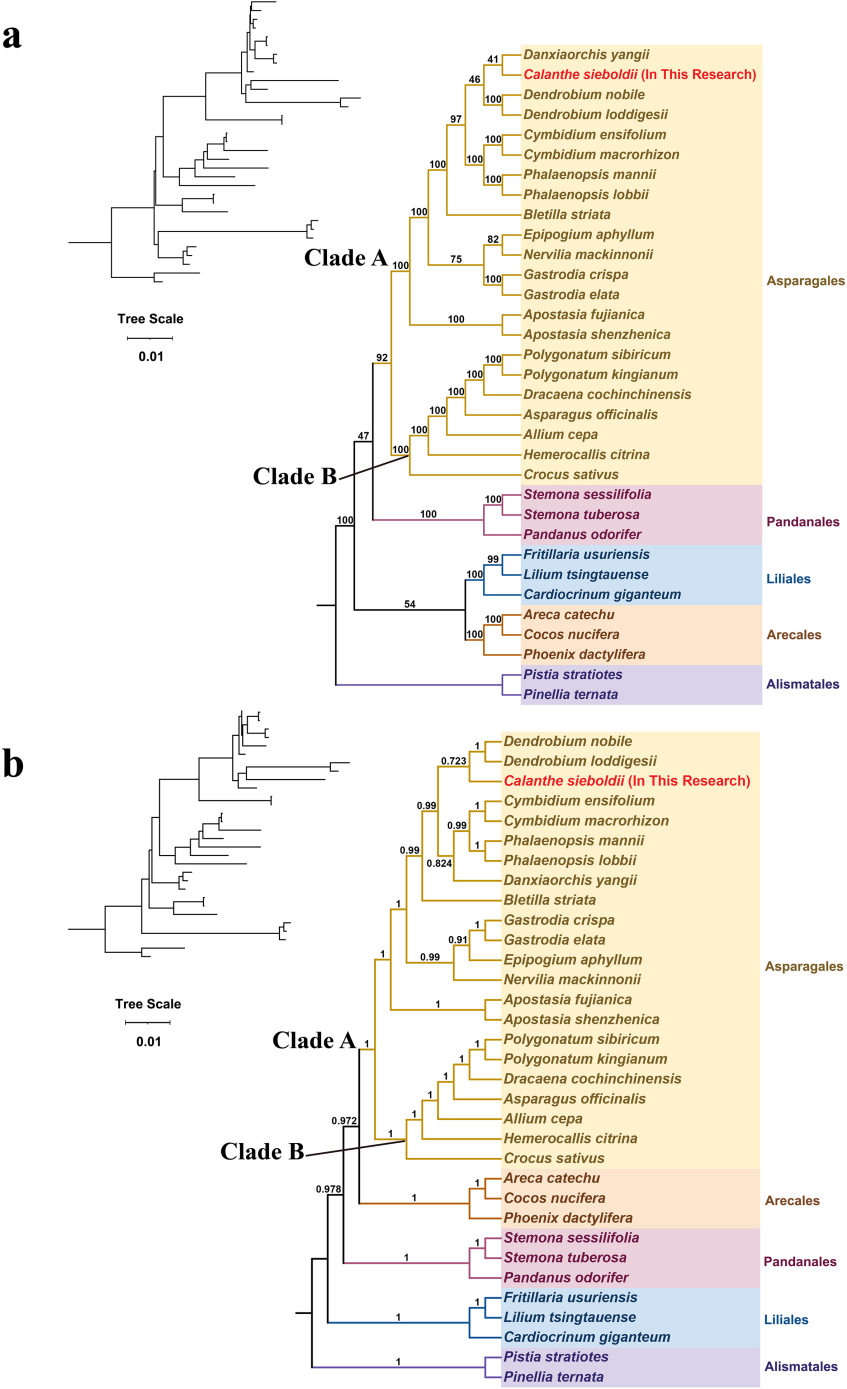
Phylogenetic placement of *Calanthe sieboldii* within the Asparagales based on mitochondrial protein-coding gene sequences. The phylogenetic trees were constructed based on a concatenated dataset of 24 conserved mitochondrial protein-coding genes from 33 angiosperm species, with two species from the order Alismatales serving as the outgroup. The position of *C. sieboldii* is highlighted in red font. **(a)** Maximum likelihood tree. Numbers at the nodes are bootstrap support values from 1,000 replicates. **(b)** Bayesian inference tree. Numbers at the nodes are posterior probabilities.

Phylogenetic analysis was conducted using a concatenated dataset of 24 conserved mitochondrial protein-coding genes from 33 species, with two species from the Alismatales included as the outgroup (**Table 3**). Both ML and BI methods produced almost identical tree topologies with strong support values (**Figure 8**). The resulting phylogenies robustly resolved the relationships among the sampled orders, with all species grouped into the expected clades of Asparagales, Pandanales, Liliales, Arecales, and Alismatales.

**Table 3:** Voucher information and GenBank accession number for taxa used in the phylogenetic analysis of 24 conserved mitochondrial protein-coding genes.

Identification	GenBank No.
*Allium cepa*	NC030100
*Apostasia fujianica*	PP724664
*Apostasia shenzhenica*	NC077647
*Areca catechu*	MW785263
*Asparagus officinalis*	NC053642
*Bletilla striata*	PQ859845-PQ859864
***Calanthe sieboldii***	**PRJNA1308661**
*Cardiocrinum giganteum*	PQ073105-PQ073123
*Cocos nucifera*	NC031696
*Crocus sativus*	OL804177
*Cymbidium ensifolium*	OR754263-OR754281
*Cymbidium macrorhizon*	OQ029542-OQ029563
*Danxiaorchis yangii*	PQ306624
*Dendrobium loddigesii*	PP829175-PP829191
*Dendrobium nobile*	OR413867-OR413891
*Dracaena ochinchinensis*	NC086589
*Epipogium aphyllum*	PQ285825-PQ285847
*Fritillaria usuriensis*	OR783162-OR783174
*Gastrodia crispa*	PP239800-PP239862
*Gastrodia elata*	PP239863-PP239927
*Hemerocallis citrina*	MZ726801-MZ726803
*Lilium tsingtauense*	OP973783-OP973810
*Nervilia mackinnonii*	PP239994-PP240051
*Pandanus odorifer*	NC080521
*Phalaenopsis lobbii*	PQ180302-PQ180319
*Phalaenopsis mannii*	PQ180326-PQ180344
*Phoenix dactylifera*	NC016740
*Pinellia ternata*	NC081910
*Pistia stratiotes*	PP530899
*Polygonatum kingianum*	PP861176-PP861177
*Polygonatum sibiricum*	PQ932596
*Stemona sessilifolia*	PP692484-PP692490
*Stemona tuberosa*	PQ374236-PQ374238

Newly generated sequences are shown in bold.

*Calanthe sieboldii* was firmly placed within the Asparagales clade (**Figure 8**). This order was resolved into two well-supported clades: Clade A (PP = 1; BS = 100%) and Clade B (PP = 1; BS = 100%). *Calanthe sieboldii* was grouped within Clade A, which exclusively comprised all 15 sampled species from the Orchidaceae. Clade B contained the remaining seven species from other Asparagales families represented (Amaryllidaceae and Asparagaceae). The topology confirmed the monophyly of the sampled orchids and the phylogenetic placement of *C. sieboldii*, consistent with large-scale phylogenomic studies of the Orchidaceae (Zhang et al., 2023; Ji et al., 2024).

## 4 Discussion

This study provides the first complete, high-resolution mitogenome assembly for *Calanthe sieboldii*, revealing a complex architecture and a suite of genomic features that are characteristic of the dynamic evolutionary history of the Orchidaceae. The assembly resolved the 644,236 bp mitogenome into 21 independent, circular chromosomes, a finding made possible by the use of long-read sequencing technology. This multipartite structure, coupled with a high density of repetitive sequences, extensive chloroplast-to-mitochondrion DNA transfer, and low synteny with other orchid genera. Together, these features reveal a genome in constant flux. These results provide a foundational genomic resource for a species of horticultural and conservation importance, and offer a detailed case study that reinforces and expands our understanding of the extreme structural plasticity that defines plant mitogenomes (Møller et al., 2021; Fan et al., 2022; Zhong et al., 2022; Jiang et al., 2023; Ni et al., 2023; Wang et al., 2023a).

The line of evidence showing *C. sieboldii* has a multicircular mitogenome is significant. It matches growing proof that plants don’t always follow the old “master circle” idea for mitochondrial DNA (Fischer et al., 2022; He et al., 2023; Bi et al., 2024). Other advanced orchids also have fragmented genomes. For example, *Dendrobium* has 20–25 chromosomes (Wang et al., 2023b; Wang et al., 2024), *Cymbidium* has 19 (Shen et al., 2024), *Paphiopedilum* has 26 (Yang et al., 2023), and *Epipogium* has 26 (Zhao et al., 2024). Even non-orchid plants like *Amorphophallus* (19 chromosomes, Shan et al., 2023) and *Punica* (7 chromosomes, Feng et al., 2023) show this pattern. This is very different from simple single-circle mitogenomes in plants like the basal orchid *Apostasia shenzhenica* (Ke et al., 2023) or the related monocot *Asparagus officinalis* (Sheng et al., 2023). Finding 21 circular chromosomes in *C. sieboldii* confirms that fragmented genomes keep popping up in orchids. This suggests genome splitting is key to how the orchid family has evolved.

This assembly gives us a solid reference, but it only comes from one *C. sieboldii* plant. We know from other plants that mitochondrial structure can vary within a species—like differences in chromosome numbers or types (Møller et al., 2021). So the exact 21-chromosome setup we found might not be the same across all *C. sieboldii* groups.

The primary driver of this structural complexity is widely considered to be repeat-mediated homologous recombination (Møller et al., 2021; Zhong et al., 2022; Ni et al., 2023). The present analysis revealed an abundance of simple and dispersed repeats, particularly tandem and palindromic sequences, distributed across all 21 chromosomes. This finding aligns perfectly with studies in genera such as *Taraxacum* (Jiang et al., 2023) and *Coffea* (Ni et al., 2023), which have experimentally validated that such repeats act as hotspots for recombination, leading to the generation of alternative genomic conformations and subgenomic circles. The abundance of these recombination-promoting elements in *C. sieboldii* provides a direct mechanistic explanation for the formation and maintenance of its complex, multicircular architecture. However, an alternative hypothesis proposed for *Thonningia* suggests that multicircular genomes may be maintained by the cessation of recombination following repeat degradation (Zhou et al., 2023). Although the present data strongly support the active recombination model, the possibility of a stabilized, low-recombination state in *C. sieboldii* presents an intriguing avenue for future investigation.

A major contributor to the size and complexity of the *C. sieboldii* mitogenome is the massive influx of chloroplast DNA. The finding that 12.72% of the *C. sieboldii* mitogenome consists of MTPTs is exceptionally high compared with most angiosperms, such as *Prunus salicina* (0.6%) (Fang et al., 2021) and *Ilex metabaptista* (2.10%) (Zhou P. et al., 2023). However, this percentage is remarkably consistent with the proportions reported in other dynamically evolving orchid mitogenomes, including *Dendrobium* (10.5%–12.0%) (Wang et al., 2023b) and *Paphiopedilum* (10.34%) (Yang et al., 2023). The discovery of 47 intact, chloroplast-derived genes within these MTPTs is particularly significant. These include numerous ribosomal protein and tRNA genes. While the retention of intact open reading frames hints at a possible role in gene replacement—similar to documented cases in other plant lineages (Chen et al., 2020)—the functional significance remains uncertain. Crucially, sequence completeness alone does not imply retained function. Without transcriptomic or proteomic evidence confirming transcription and translation within mitochondria, these transferred sequences could merely represent structurally preserved pseudogenes rather than functional genes. Thus, their biological relevance requires direct experimental validation. Nevertheless, The scale of this interorganellar gene flow in *C. sieboldii* underscores its role as a major evolutionary force shaping the content and structure of orchid mitogenomes.

The highly dynamic nature of the *C. sieboldii* mitogenome is further emphasized by the synteny analysis, which revealed an almost complete lack of gene-order conservation compared with other orchids. This low collinearity is a direct genomic signature of the frequent fragmentation, recombination, and inversion events that have shuffled the genome over evolutionary time. This genomic instability provides a potential molecular basis for the rapid speciation and adaptive radiation that characterize the Orchidaceae (Jacquemyn et al., 2023; Zhang et al., 2023). The taxonomic complexity and polyphyly within the *Calanthe* alliance, as documented by Zhai et al (Zhai et al., 2014), are the macroscopic outcomes of the microscopic genomic volatility we have observed here. The highly rearranged genome of *C. sieboldii* is an exemplar of the “rapid structural evolution, slow sequence evolution” paradox that characterizes plant mitogenomes (Fan et al., 2022).

Despite this dramatic structural flux, core molecular functions appear to be under stabilizing selection. The predicted patterns of RNA editing and codon usage in *C. sieboldii* are highly conserved and consistent with those observed in a wide range of angiosperms. Our computational analysis suggested approximately 574 C-to-U RNA editing sites. However, it must be noted that in silico prediction tools, despite their utility, are prone to false positives and may not fully capture tissue-specific editing dynamics or the influence of nuclear-encoded factors (Edera et al., 2021). Therefore, although transcriptomic sequencing is required to verify specific editing events, the overall abundance is comparable to that reported in other complex mitogenomes (Yang et al., 2023; Han et al., 2024; Liu et al., 2024). More importantly, the genomic distribution of these putative sites—with hotspots concentrated in the functionally essential *nad* and *ccm* gene families and very few sites in ribosomal protein genes, closely mirrors the patterns found in diverse genera such as *Trigonella* (He et al., 2023) and *Photinia* [11]. This suggests a conserved, fine-tuned regulatory mechanism that ensures the functional integrity of core bioenergetic protein complexes, a process likely mediated by a large family of nuclear gene-encoded Pentatricopeptide Repeat proteins [10]. Similarly, the strong preference for A/U at the third codon position is a common feature of plant mitogenomes, reflecting a shared mutational bias or selective pressure across angiosperms [51]. This conservation of core molecular processes highlights that, although the architecture of the *C. sieboldii* mitogenome is exceptionally plastic, its fundamental role in cellular respiration is maintained by robust and conserved post-transcriptional and translational mechanisms.

This study provides the first complete, annotated, multichromosomal reference mitogenome for *Calanthe sieboldii*, a notable contribution given the horticultural importance and endangered status of the genus [22, 61]. This work definitively characterizes its complex genomic architecture and quantifies the critical features—high repeat content, massive MTPT integration, and low synteny—that are hallmarks of the rapid and dynamic evolution of orchid mitogenomes. However, this study has several limitations. As it is based on a single individual, it does not capture the potential for intraspecific structural variation. Furthermore, recombination activity of the identified repeats and the functional status of the transferred chloroplast genes are inferred from sequence data and have not been experimentally validated. Future research should focus on population-level mitogenomics to assess structural polymorphism within *C. sieboldii*. Transcriptomic analysis (RNA-sequencing) would be invaluable for experimental validation of the predicted RNA editing sites and determining whether the 47 intact chloroplast-derived genes are expressed within the mitochondrion. Finally, investigating the nuclear genome for the status of DNA replication, recombination, and repair (DNA-RRR) genes, as suggested by studies on other multipartite genomes [56, 62], could provide deeper insights into the mechanisms that permit such extreme genomic fragmentation.

## 5 Conclusion

In conclusion, this research dissects the intricate mitochondrial genome of *Calanthe sieboldii*, revealing a highly fragmented structure with 21 chromosomes shaped by extensive repeat-mediated recombination and a massive influx of chloroplast DNA. These findings provide a detailed genomic snapshot that reinforces the paradigm of extreme structural plasticity in Orchidaceae mitogenomes. The high-quality, annotated genome presented here serves as a vital public resource that will facilitate future research into the evolution, population genetics, and conservation of the diverse and horticulturally valuable *Calanthe* genus.

## Data Availability

The datasets presented in this study can be found in online repositories. The names of the repository/repositories and accession number(s) can be found below: https://www.ncbi.nlm.nih.gov/genbank/, PRJNA1308661.
