# Supplementary material for: Assembly and comparative analysis of the complete multichromosomal mitochondrial genome of an endangered orchid species, Calanthe sieboldii
Source: Front Plant Sci. 2026 Mar 10;17:1701753. doi: 10.3389/fpls.2026.1701753 (PMC13008936; doi:10.3389/fpls.2026.1701753)
Supplement: Supplementary file 1 [file DataSheet1.docx]

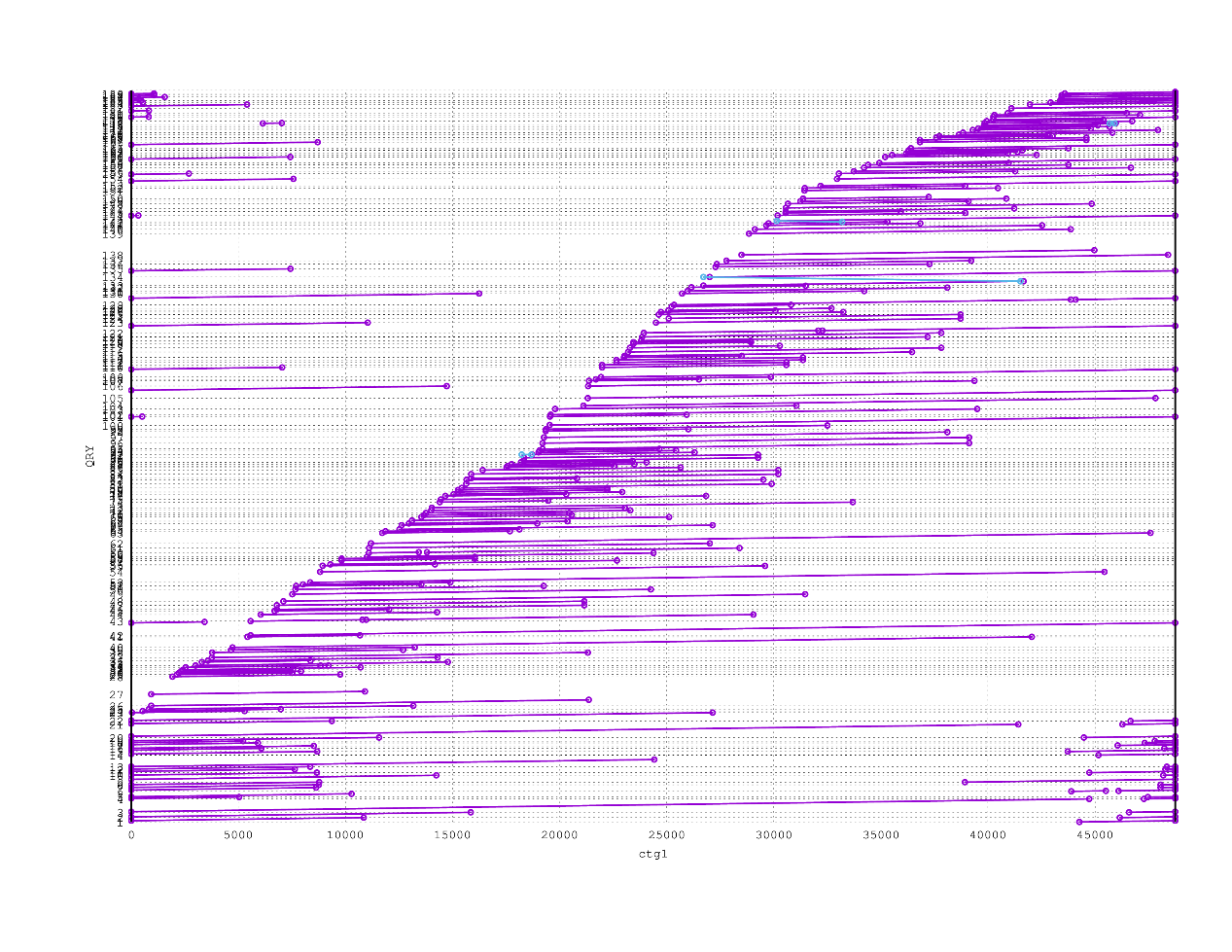


**Supplementary Figure S1. Long-read validation of the circular structure of mitochondrial contig 1.** The dot plot visualization displays the alignment of Oxford Nanopore Technologies (ONT) long reads against the assembled mitochondrial contig 1 (ctg1). The x-axis represents the reference contig sequence, and the y-axis represents individual query reads. The continuous diagonal alignments (purple lines) covering the entire length of the contig provide robust evidence for the assembly's continuity and confirm the genuine circular nature of the molecule.


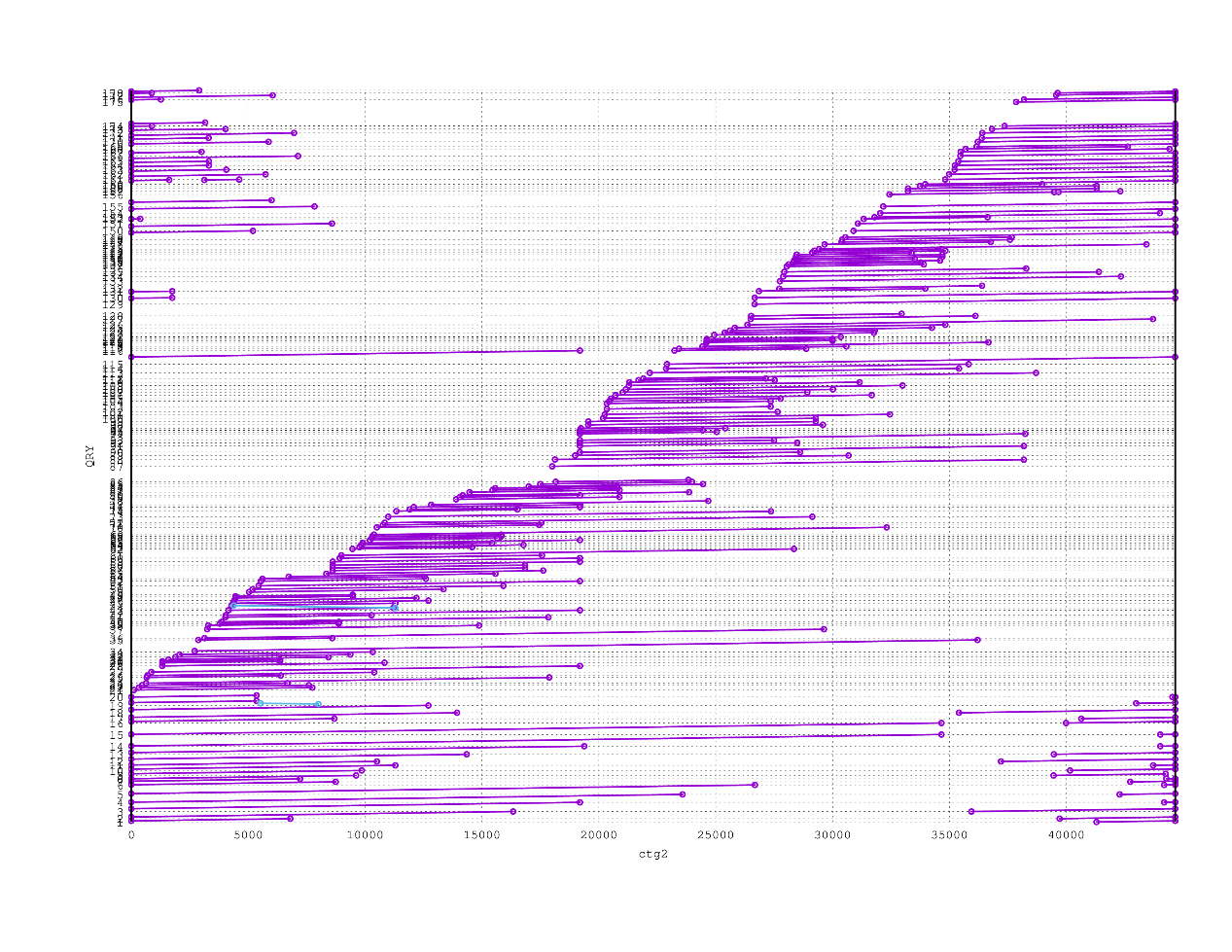


**Supplementary Figure S2. Long-read validation of the circular structure of mitochondrial contig 2.** The dot plot visualization displays the alignment of Oxford Nanopore Technologies (ONT) long reads against the assembled mitochondrial contig 2 (ctg2). The x-axis represents the reference contig sequence, and the y-axis represents individual query reads. The continuous diagonal alignments (purple lines) covering the entire length of the contig provide robust evidence for the assembly's continuity and confirm the genuine circular nature of the molecule.


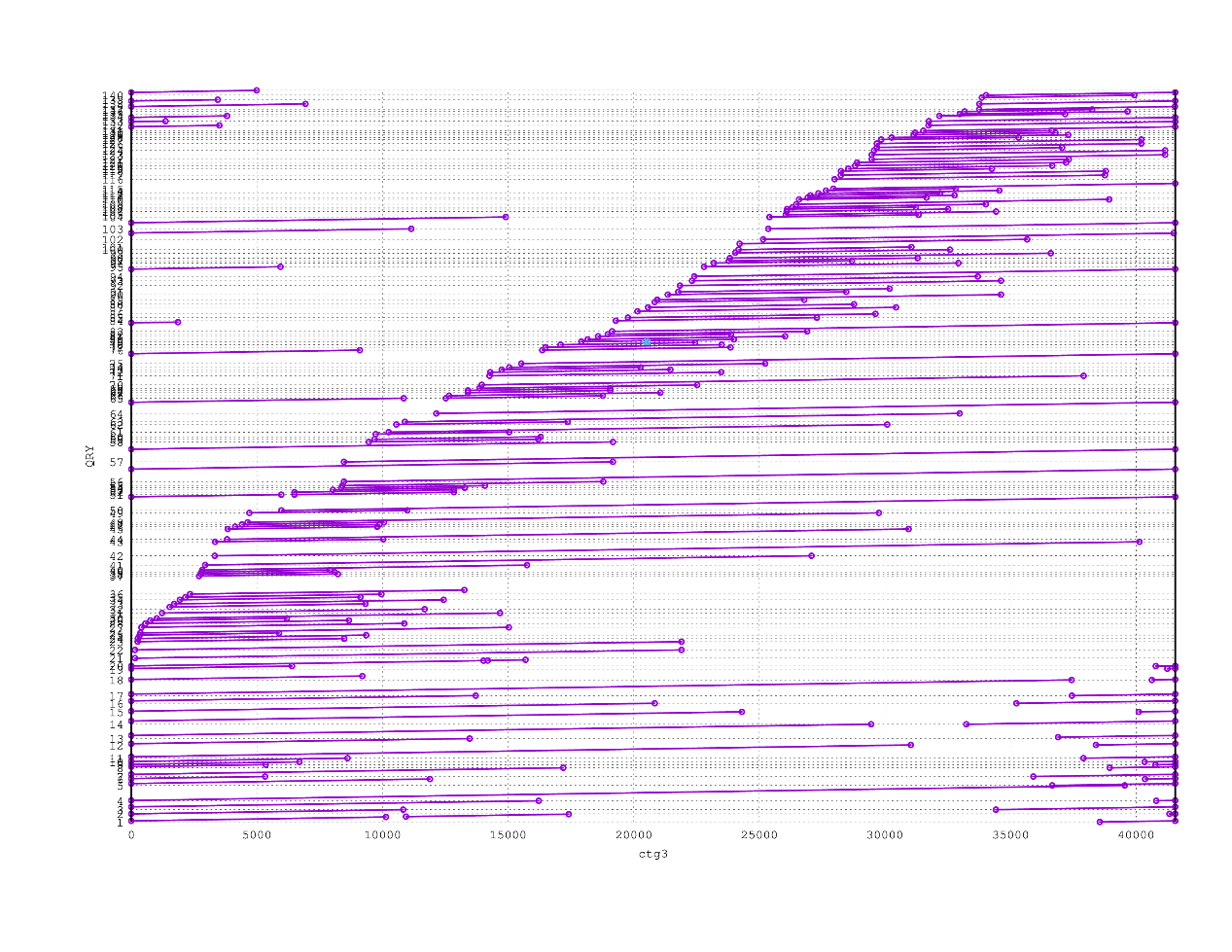


**Supplementary Figure S3. Long-read validation of the circular structure of mitochondrial contig 3.** The dot plot visualization displays the alignment of Oxford Nanopore Technologies (ONT) long reads against the assembled mitochondrial contig 3 (ctg3). The x-axis represents the reference contig sequence, and the y-axis represents individual query reads. The continuous diagonal alignments (purple lines) covering the entire length of the contig provide robust evidence for the assembly's continuity and confirm the genuine circular nature of the molecule.


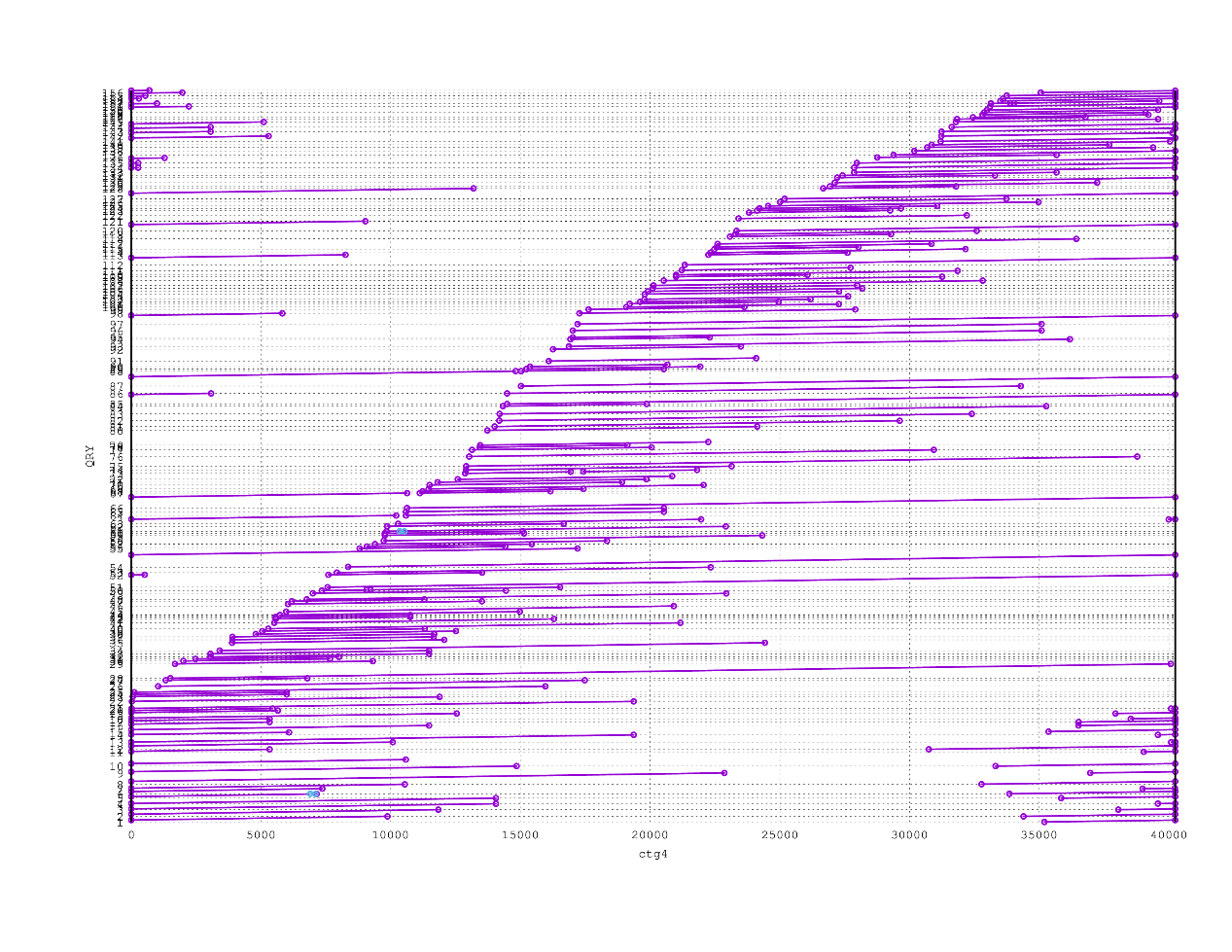


**Supplementary Figure S4. Long-read validation of the circular structure of mitochondrial contig 4.** The dot plot visualization displays the alignment of Oxford Nanopore Technologies (ONT) long reads against the assembled mitochondrial contig 4 (ctg4). The x-axis represents the reference contig sequence, and the y-axis represents individual query reads. The continuous diagonal alignments (purple lines) covering the entire length of the contig provide robust evidence for the assembly's continuity and confirm the genuine circular nature of the molecule.


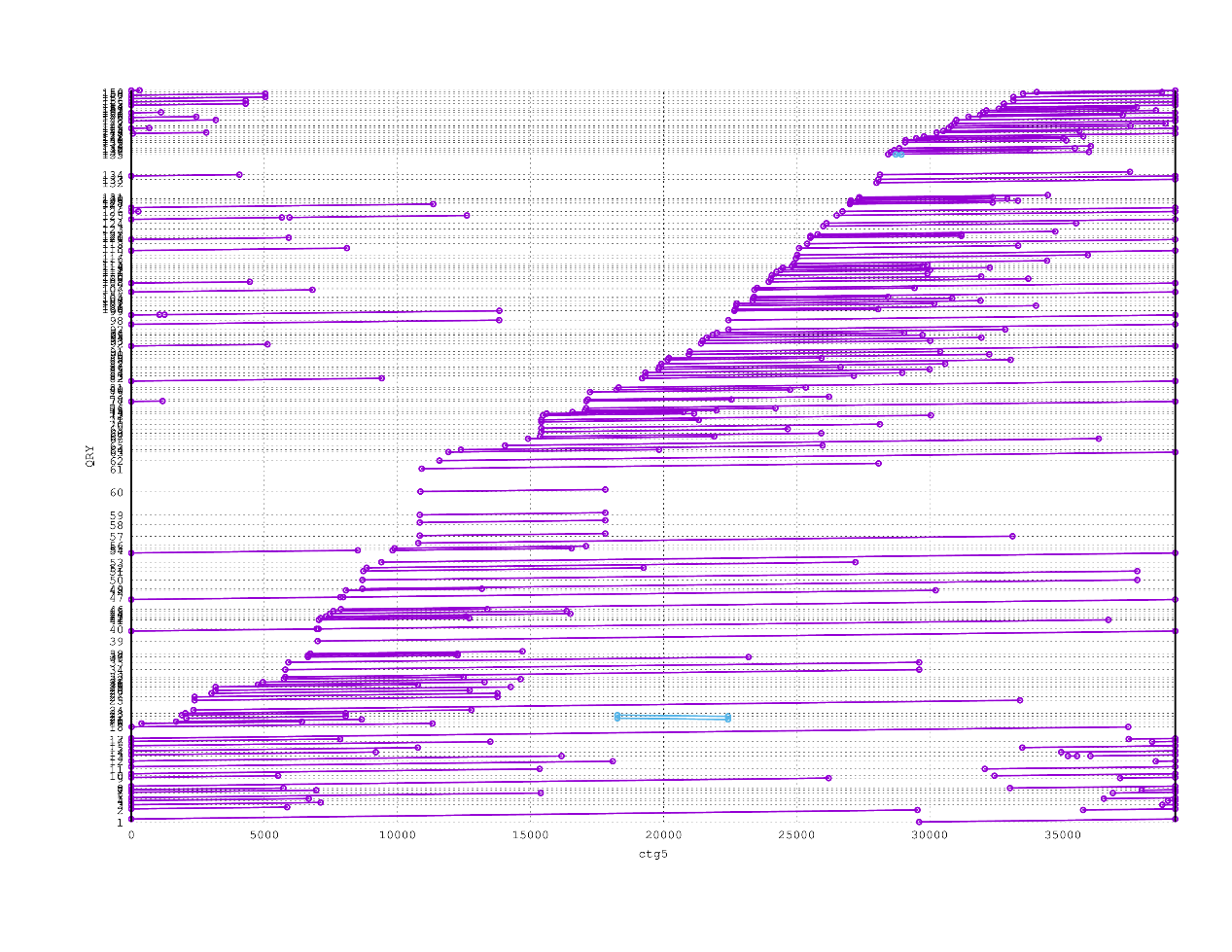


**Supplementary Figure S5. Long-read validation of the circular structure of mitochondrial contig 5.** The dot plot visualization displays the alignment of Oxford Nanopore Technologies (ONT) long reads against the assembled mitochondrial contig 5 (ctg5). The x-axis represents the reference contig sequence, and the y-axis represents individual query reads. The continuous diagonal alignments (purple lines) covering the entire length of the contig provide robust evidence for the assembly's continuity and confirm the genuine circular nature of the molecule.


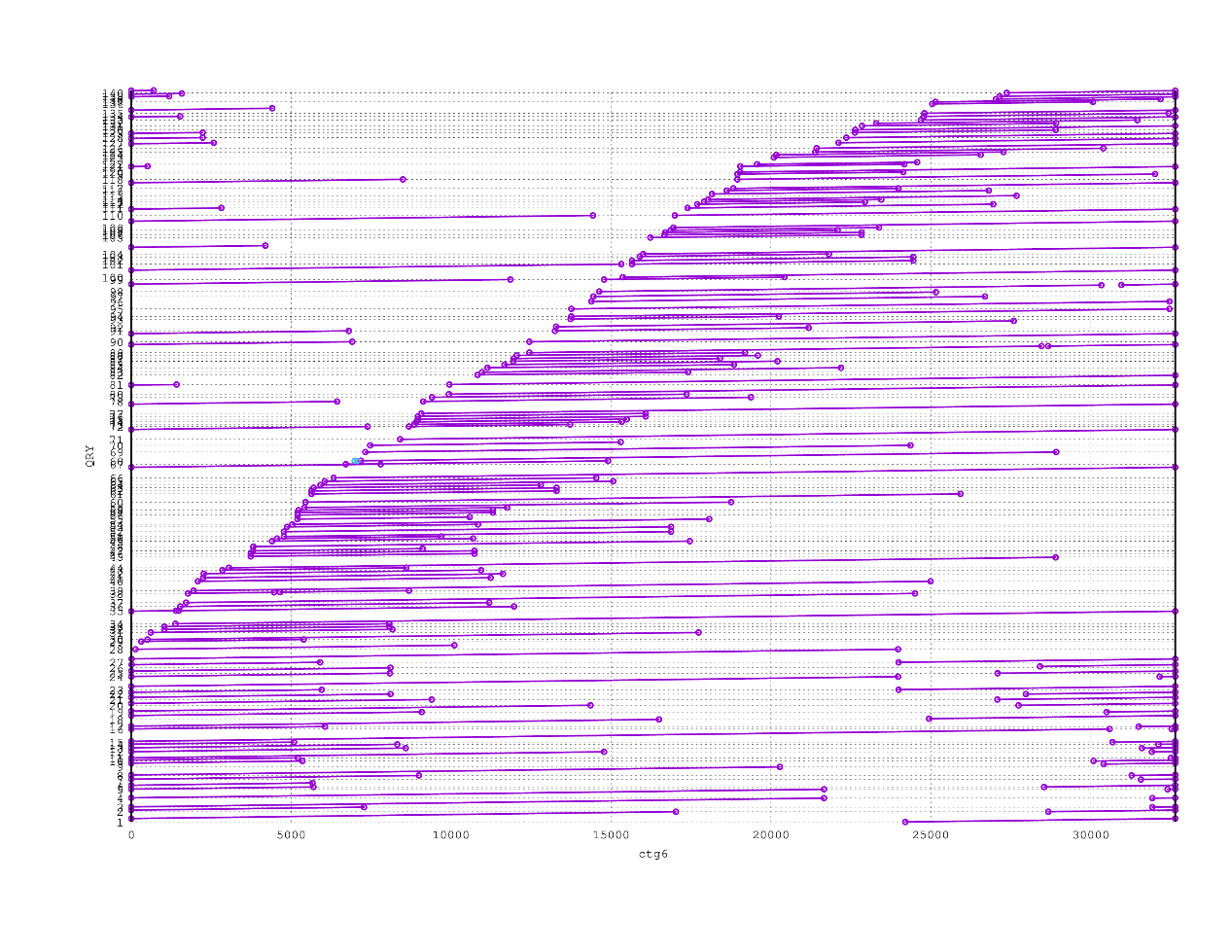


**Supplementary Figure S6. Long-read validation of the circular structure of mitochondrial contig 6.** The dot plot visualization displays the alignment of Oxford Nanopore Technologies (ONT) long reads against the assembled mitochondrial contig 6 (ctg6). The x-axis represents the reference contig sequence, and the y-axis represents individual query reads. The continuous diagonal alignments (purple lines) covering the entire length of the contig provide robust evidence for the assembly's continuity and confirm the genuine circular nature of the molecule.


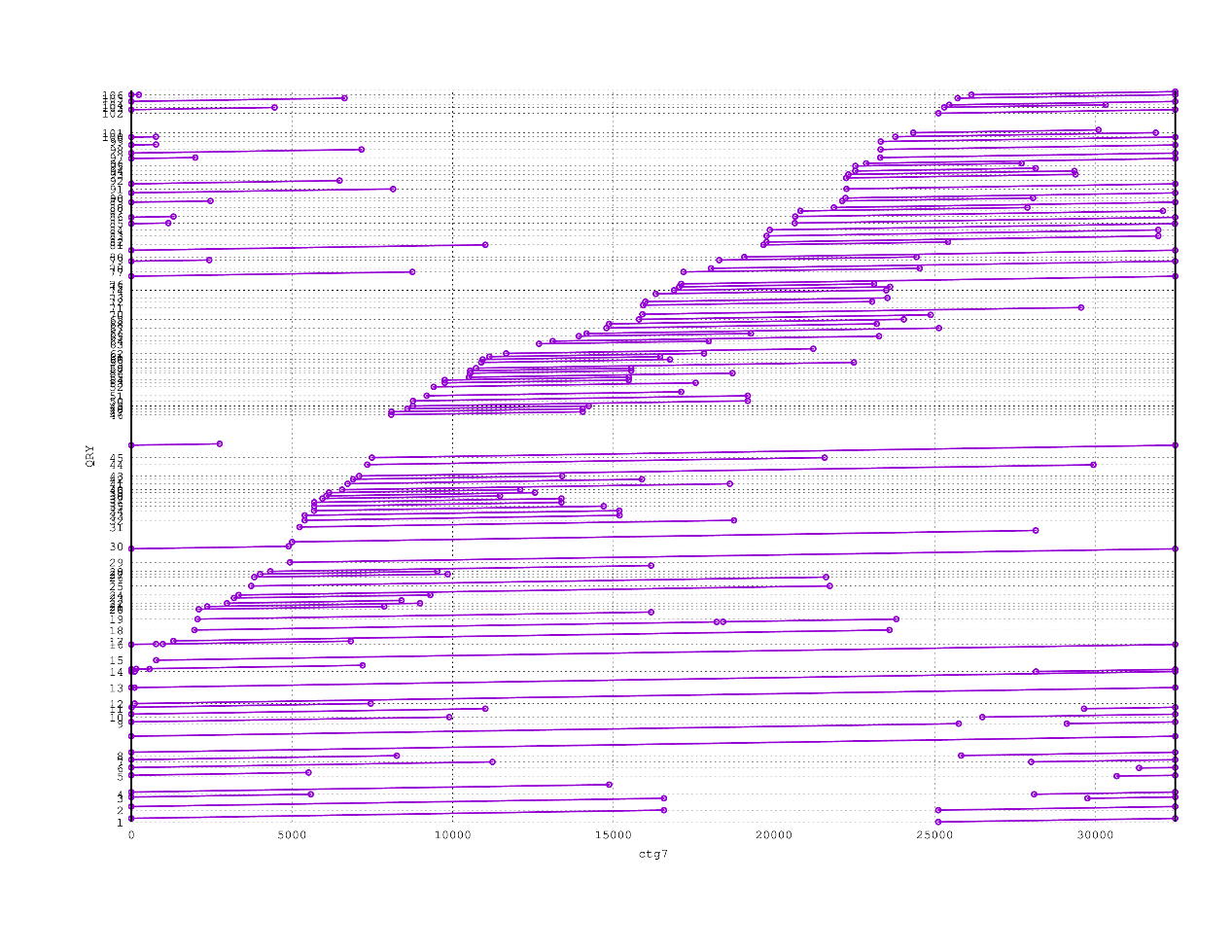


**Supplementary Figure S7. Long-read validation of the circular structure of mitochondrial contig 7.** The dot plot visualization displays the alignment of Oxford Nanopore Technologies (ONT) long reads against the assembled mitochondrial contig 7 (ctg7). The x-axis represents the reference contig sequence, and the y-axis represents individual query reads. The continuous diagonal alignments (purple lines) covering the entire length of the contig provide robust evidence for the assembly's continuity and confirm the genuine circular nature of the molecule.


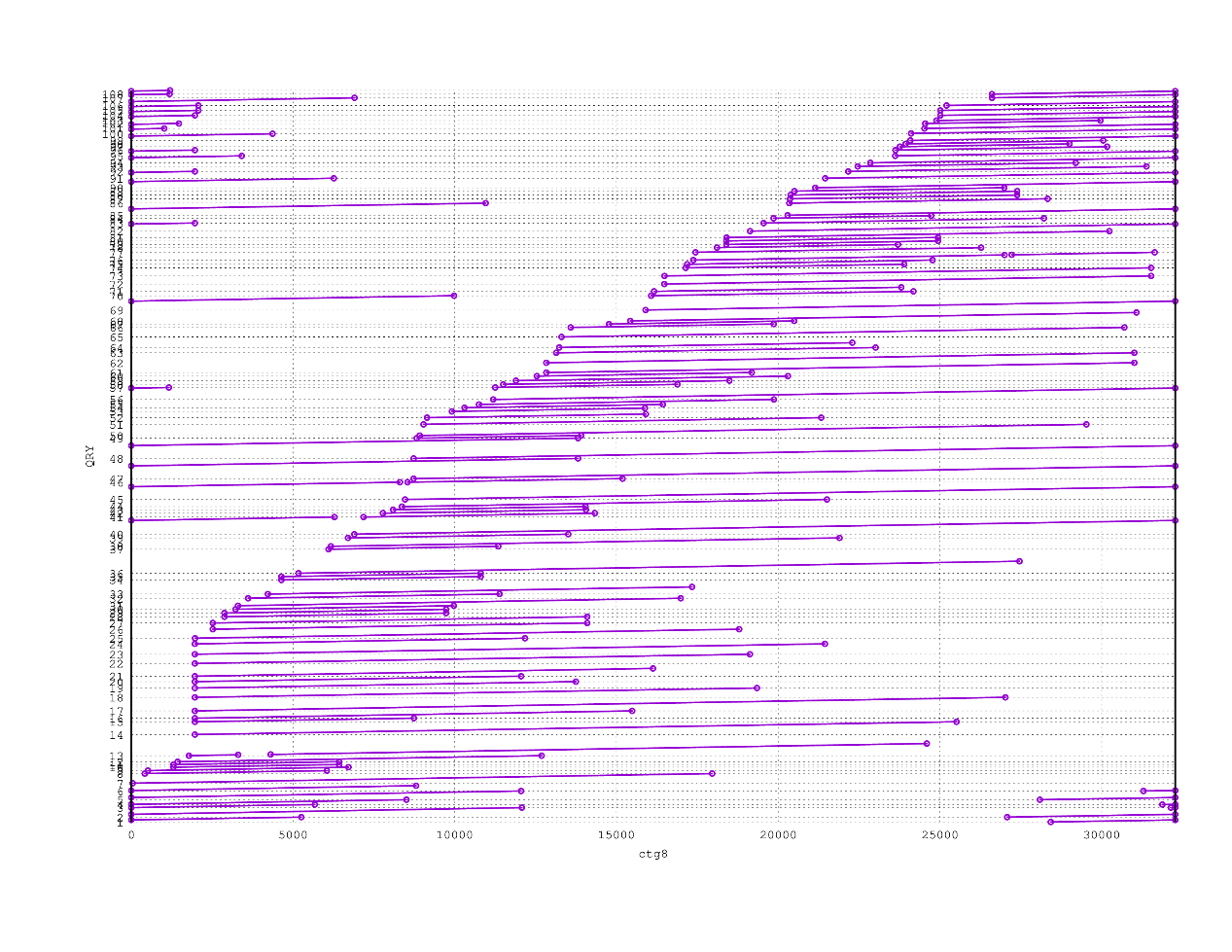


**Supplementary Figure S8. Long-read validation of the circular structure of mitochondrial contig 8.** The dot plot visualization displays the alignment of Oxford Nanopore Technologies (ONT) long reads against the assembled mitochondrial contig 8 (ctg8). The x-axis represents the reference contig sequence, and the y-axis represents individual query reads. The continuous diagonal alignments (purple lines) covering the entire length of the contig provide robust evidence for the assembly's continuity and confirm the genuine circular nature of the molecule.


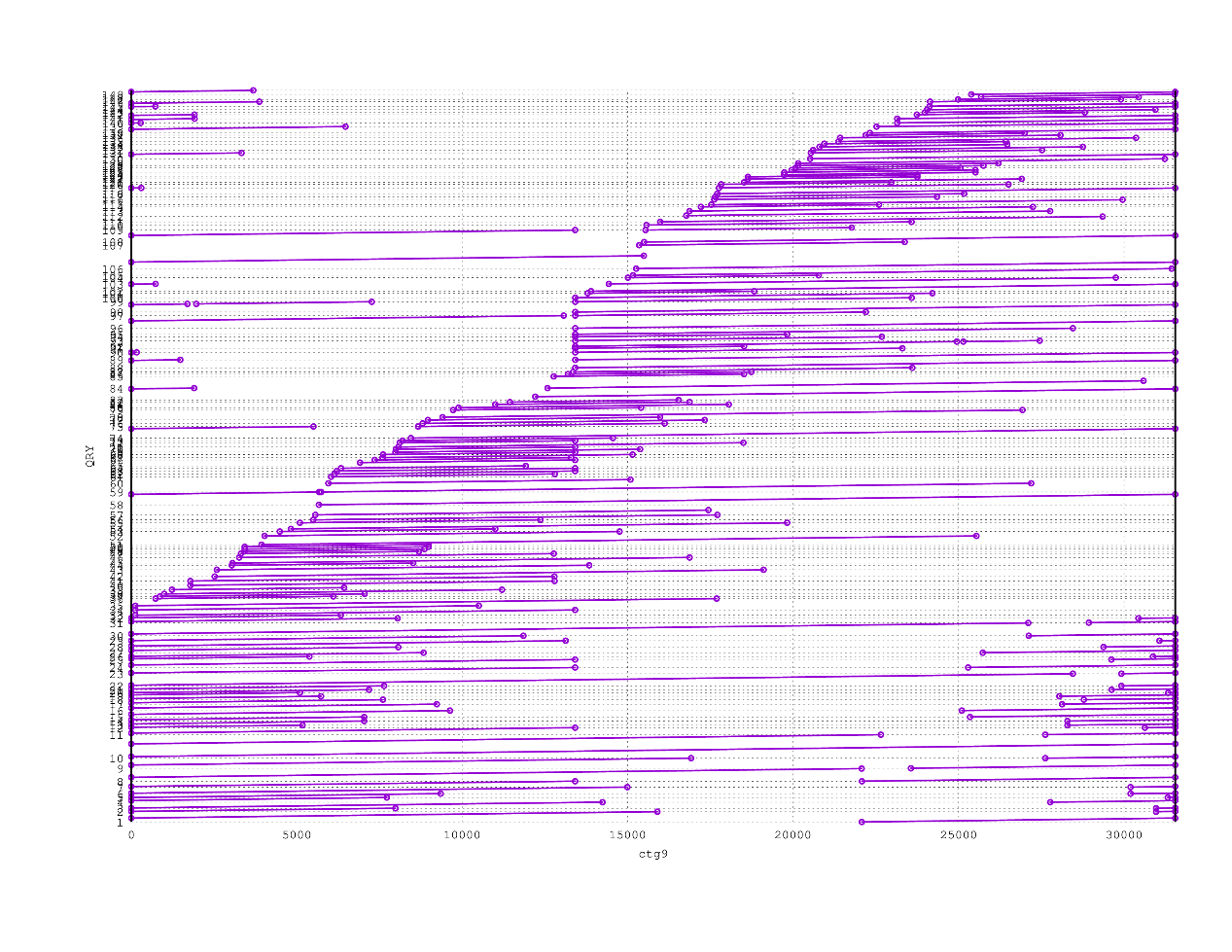


**Supplementary Figure S9. Long-read validation of the circular structure of mitochondrial contig 9.** The dot plot visualization displays the alignment of Oxford Nanopore Technologies (ONT) long reads against the assembled mitochondrial contig 9 (ctg9). The x-axis represents the reference contig sequence, and the y-axis represents individual query reads. The continuous diagonal alignments (purple lines) covering the entire length of the contig provide robust evidence for the assembly's continuity and confirm the genuine circular nature of the molecule.


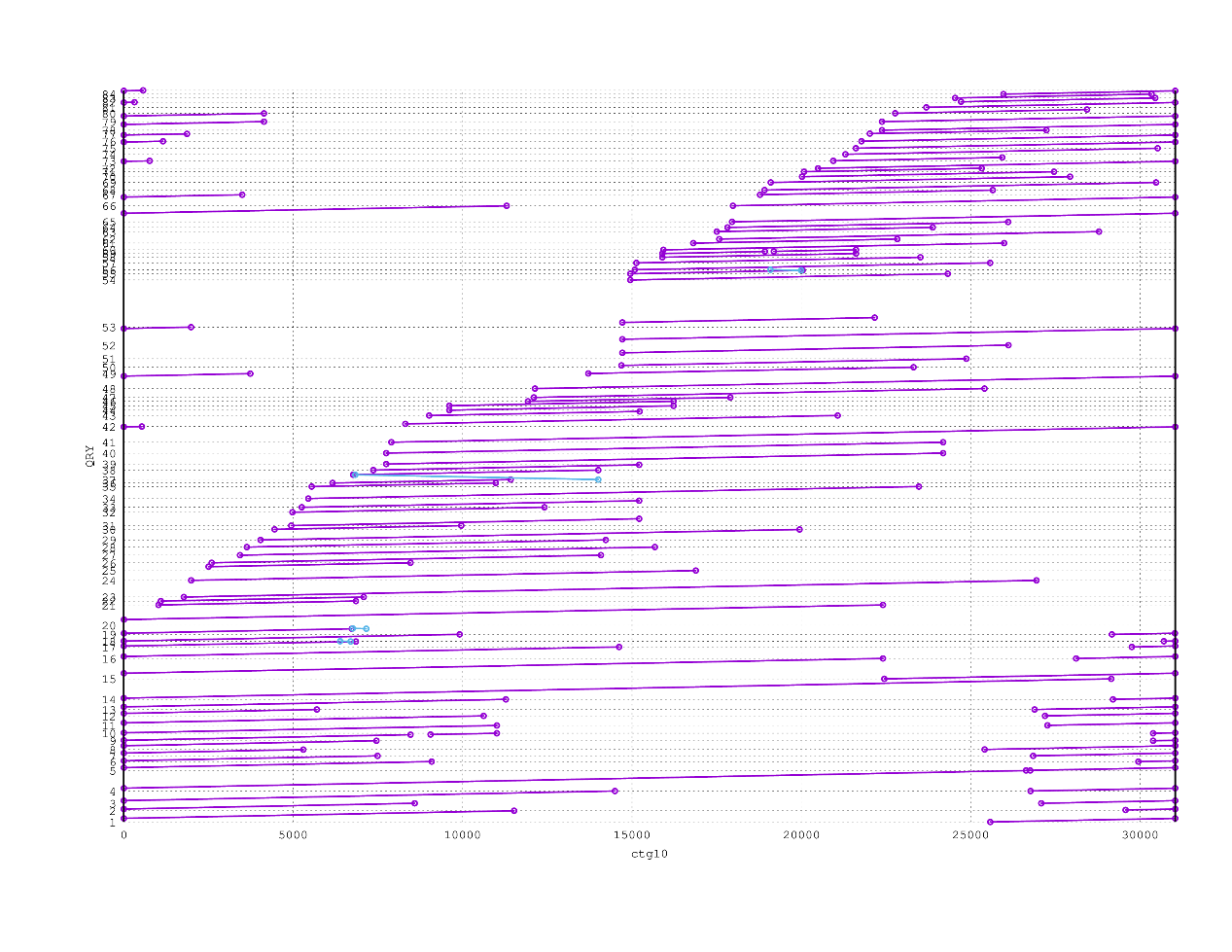


**Supplementary Figure S10. Long-read validation of the circular structure of mitochondrial contig 10.** The dot plot visualization displays the alignment of Oxford Nanopore Technologies (ONT) long reads against the assembled mitochondrial contig 10 (ctg10). The x-axis represents the reference contig sequence, and the y-axis represents individual query reads. The continuous diagonal alignments (purple lines) covering the entire length of the contig provide robust evidence for the assembly's continuity and confirm the genuine circular nature of the molecule.


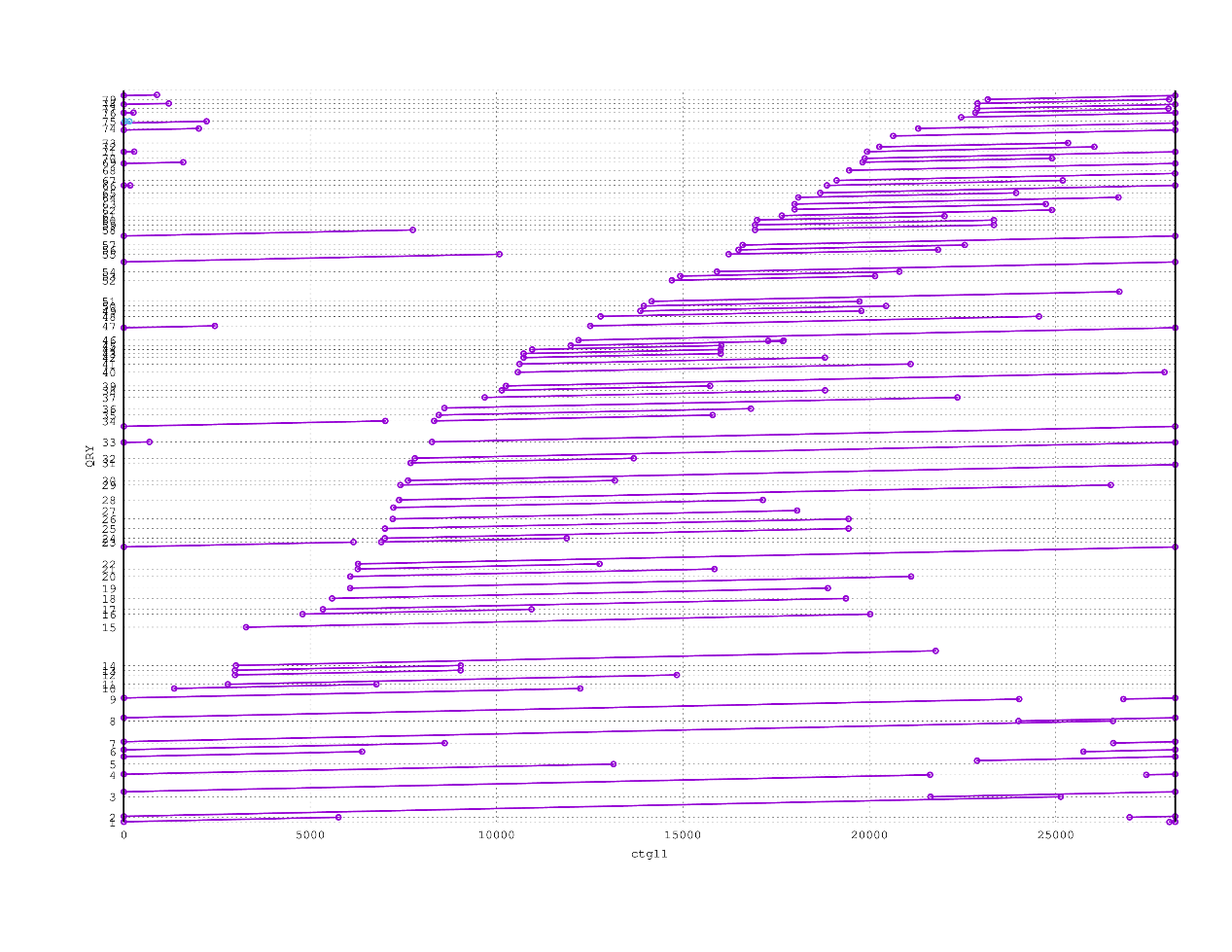


**Supplementary Figure S11. Long-read validation of the circular structure of mitochondrial contig 11.** The dot plot visualization displays the alignment of Oxford Nanopore Technologies (ONT) long reads against the assembled mitochondrial contig 11 (ctg11). The x-axis represents the reference contig sequence, and the y-axis represents individual query reads. The continuous diagonal alignments (purple lines) covering the entire length of the contig provide robust evidence for the assembly's continuity and confirm the genuine circular nature of the molecule.


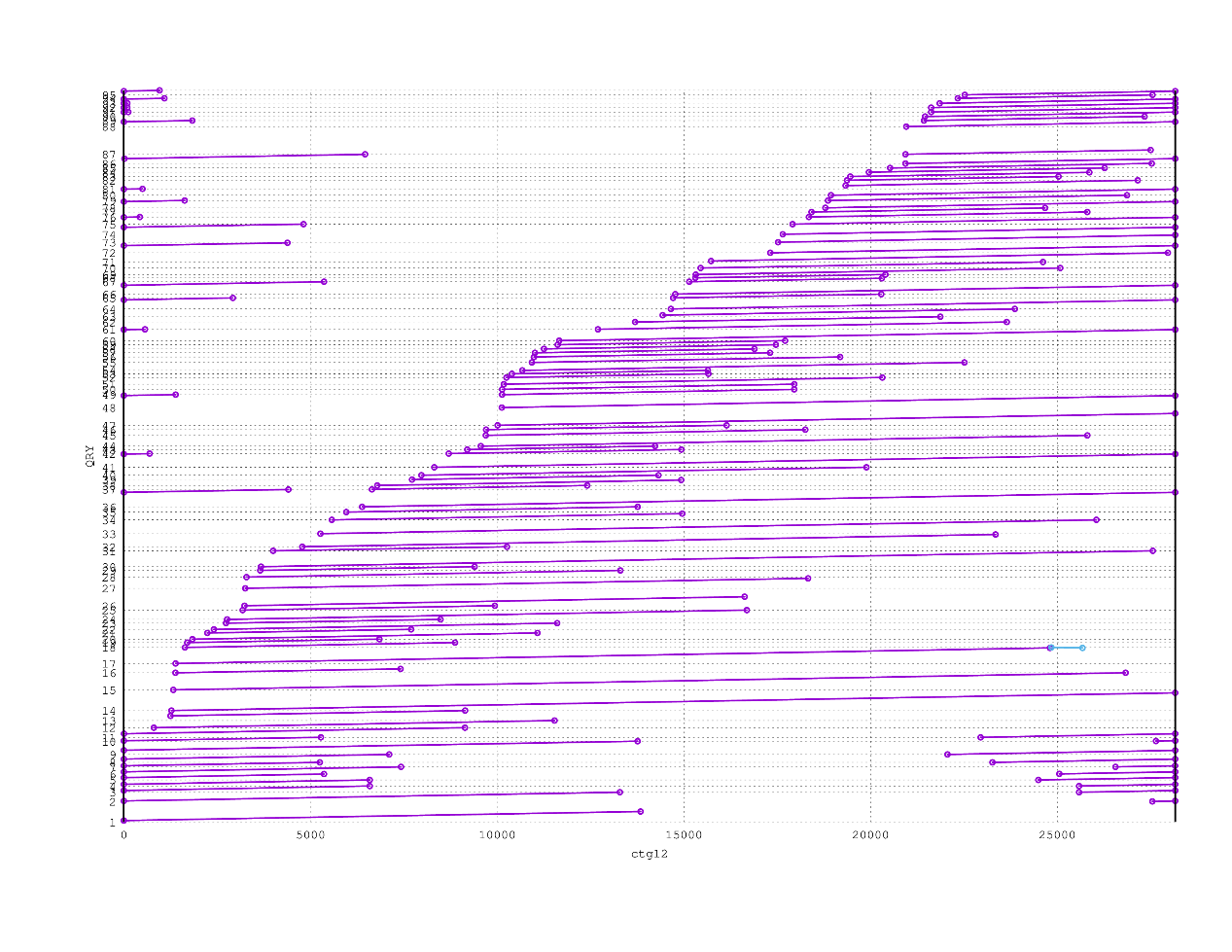


**Supplementary Figure S12. Long-read validation of the circular structure of mitochondrial contig 12.** The dot plot visualization displays the alignment of Oxford Nanopore Technologies (ONT) long reads against the assembled mitochondrial contig 12 (ctg12). The x-axis represents the reference contig sequence, and the y-axis represents individual query reads. The continuous diagonal alignments (purple lines) covering the entire length of the contig provide robust evidence for the assembly's continuity and confirm the genuine circular nature of the molecule.


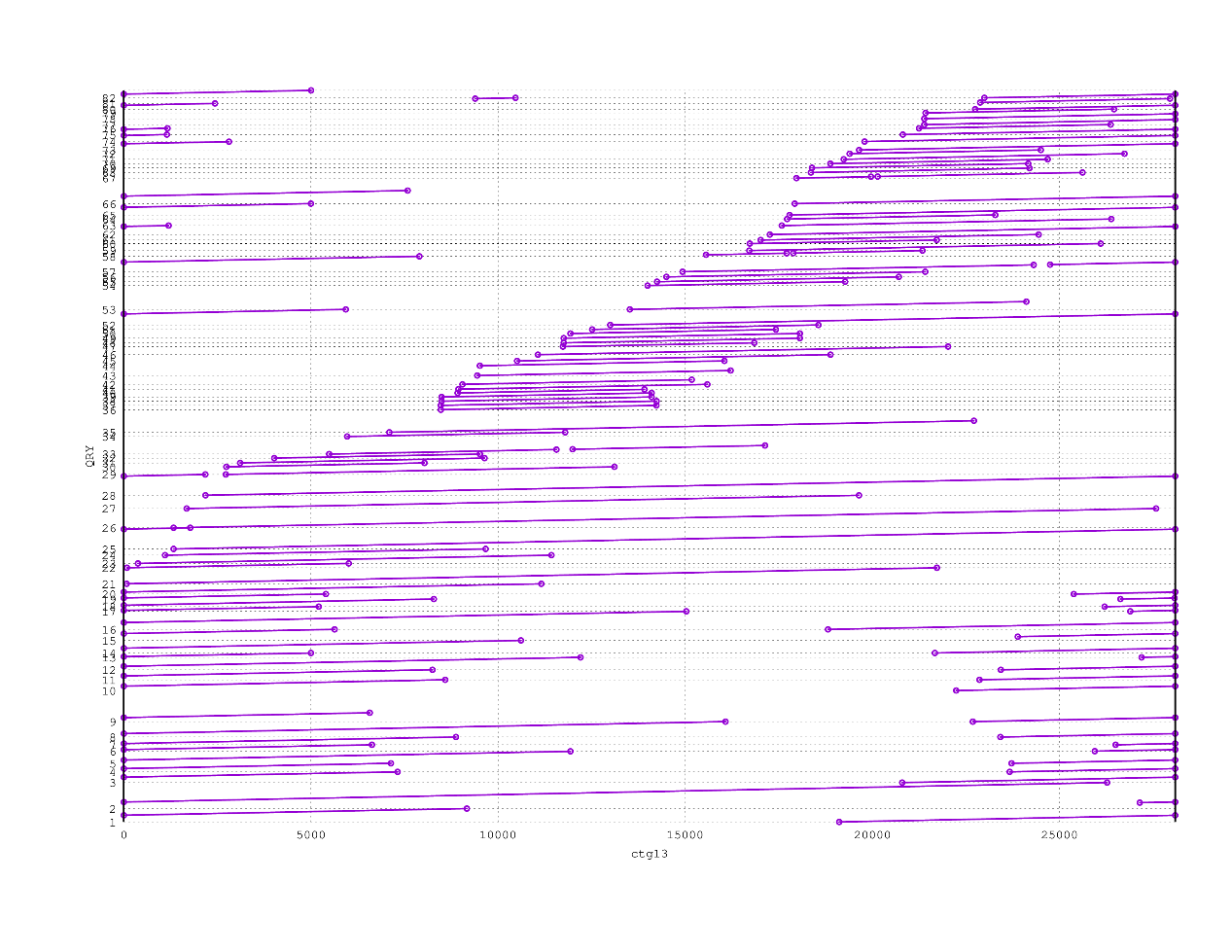


**Supplementary Figure S13. Long-read validation of the circular structure of mitochondrial contig 13.** The dot plot visualization displays the alignment of Oxford Nanopore Technologies (ONT) long reads against the assembled mitochondrial contig 13 (ctg13). The x-axis represents the reference contig sequence, and the y-axis represents individual query reads. The continuous diagonal alignments (purple lines) covering the entire length of the contig provide robust evidence for the assembly's continuity and confirm the genuine circular nature of the molecule.


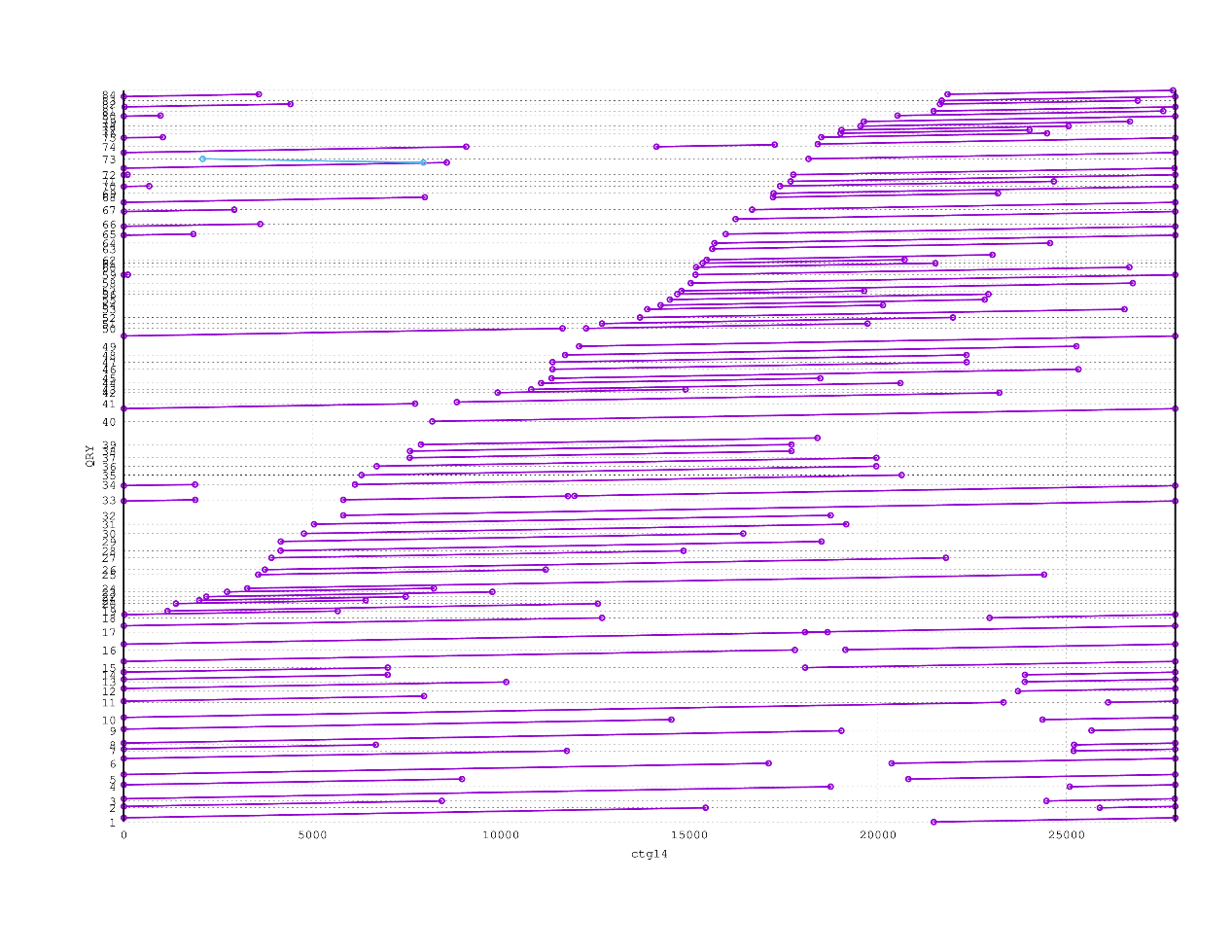


**Supplementary Figure S14. Long-read validation of the circular structure of mitochondrial contig 14.** The dot plot visualization displays the alignment of Oxford Nanopore Technologies (ONT) long reads against the assembled mitochondrial contig 14 (ctg14). The x-axis represents the reference contig sequence, and the y-axis represents individual query reads. The continuous diagonal alignments (purple lines) covering the entire length of the contig provide robust evidence for the assembly's continuity and confirm the genuine circular nature of the molecule.


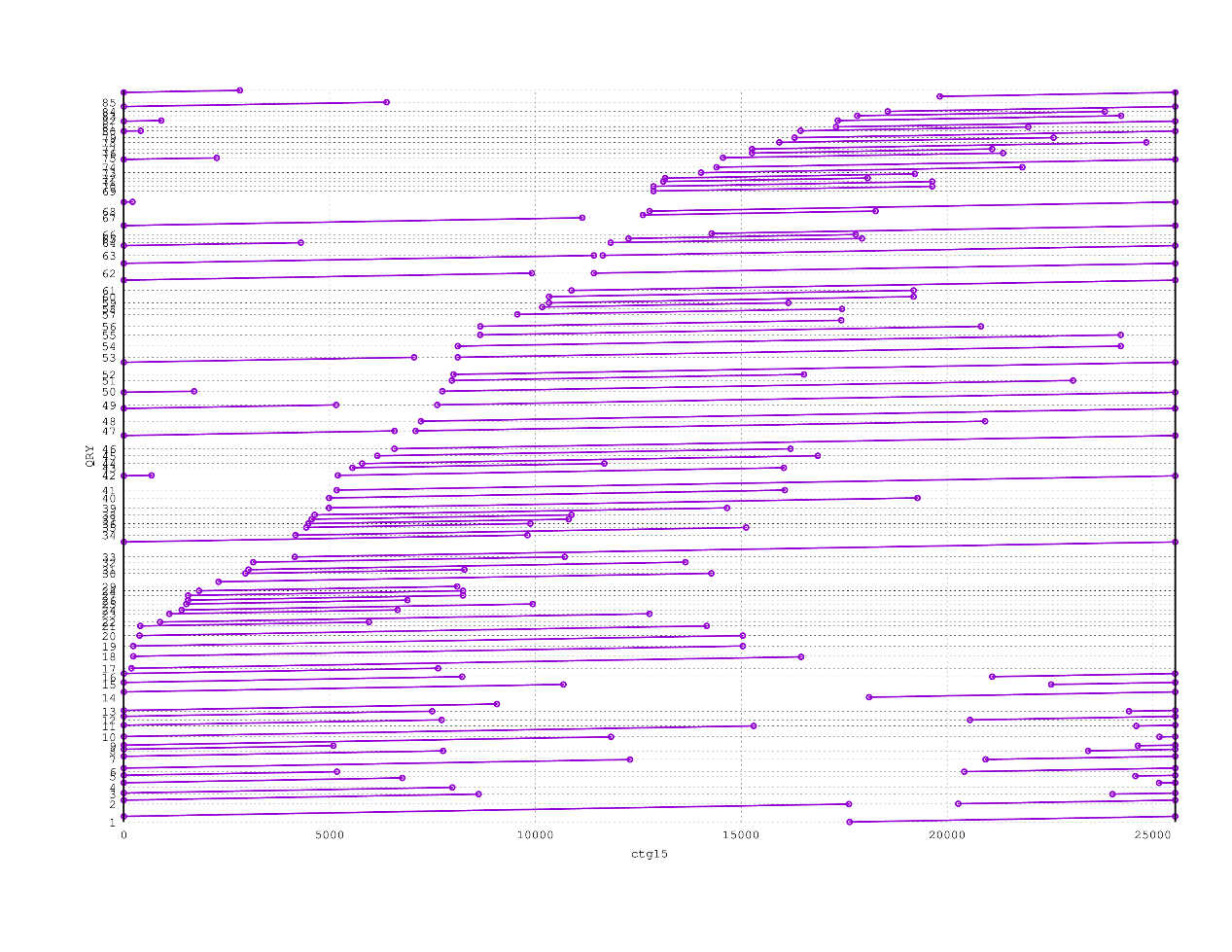


**Supplementary Figure S15. Long-read validation of the circular structure of mitochondrial contig 15.** The dot plot visualization displays the alignment of Oxford Nanopore Technologies (ONT) long reads against the assembled mitochondrial contig 15 (ctg15). The x-axis represents the reference contig sequence, and the y-axis represents individual query reads. The continuous diagonal alignments (purple lines) covering the entire length of the contig provide robust evidence for the assembly's continuity and confirm the genuine circular nature of the molecule.


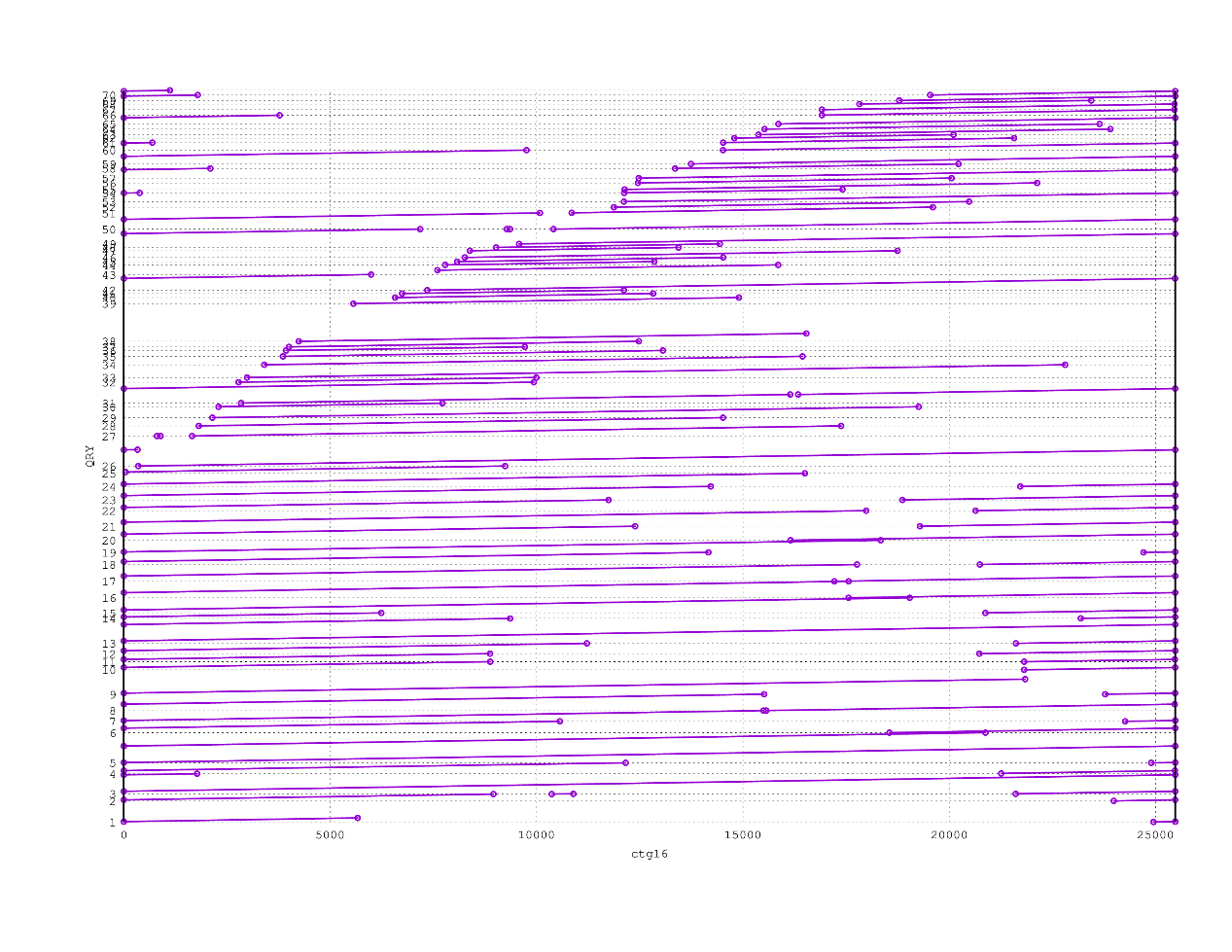


**Supplementary Figure S16. Long-read validation of the circular structure of mitochondrial contig 16.** The dot plot visualization displays the alignment of Oxford Nanopore Technologies (ONT) long reads against the assembled mitochondrial contig 16 (ctg16). The x-axis represents the reference contig sequence, and the y-axis represents individual query reads. The continuous diagonal alignments (purple lines) covering the entire length of the contig provide robust evidence for the assembly's continuity and confirm the genuine circular nature of the molecule.


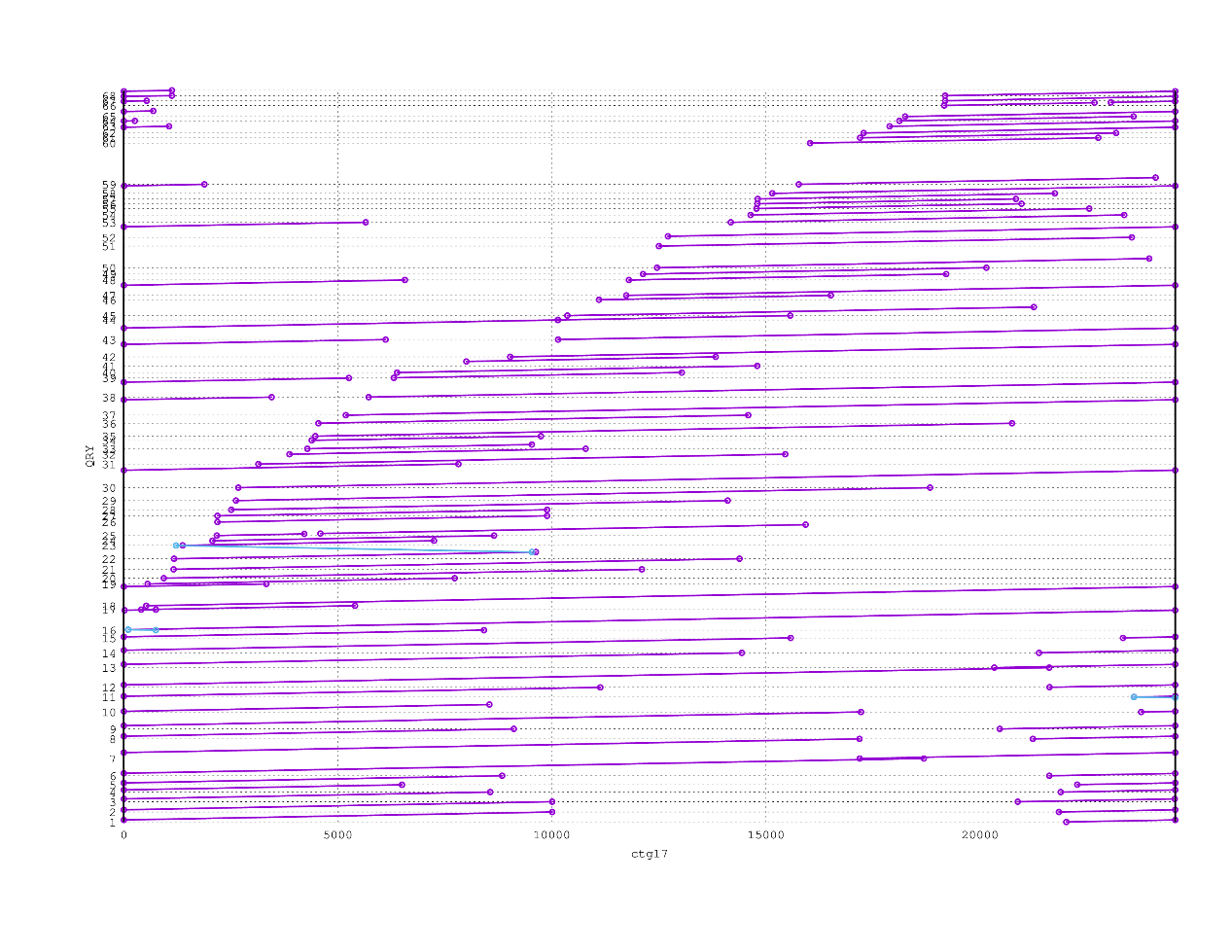


**Supplementary Figure S17. Long-read validation of the circular structure of mitochondrial contig 17.** The dot plot visualization displays the alignment of Oxford Nanopore Technologies (ONT) long reads against the assembled mitochondrial contig 17 (ctg17). The x-axis represents the reference contig sequence, and the y-axis represents individual query reads. The continuous diagonal alignments (purple lines) covering the entire length of the contig provide robust evidence for the assembly's continuity and confirm the genuine circular nature of the molecule.


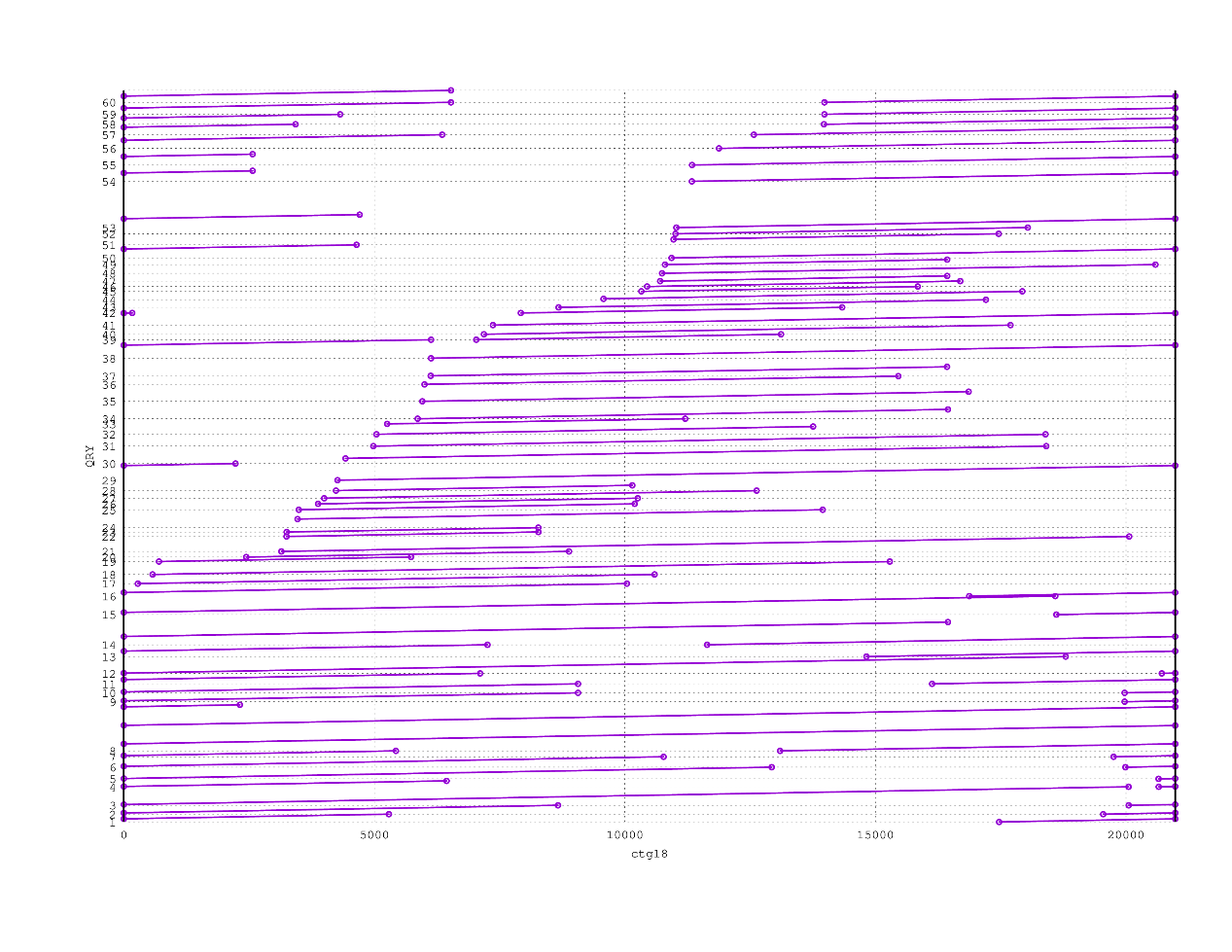


**Supplementary Figure S18. Long-read validation of the circular structure of mitochondrial contig 18.** The dot plot visualization displays the alignment of Oxford Nanopore Technologies (ONT) long reads against the assembled mitochondrial contig 18 (ctg18). The x-axis represents the reference contig sequence, and the y-axis represents individual query reads. The continuous diagonal alignments (purple lines) covering the entire length of the contig provide robust evidence for the assembly's continuity and confirm the genuine circular nature of the molecule.


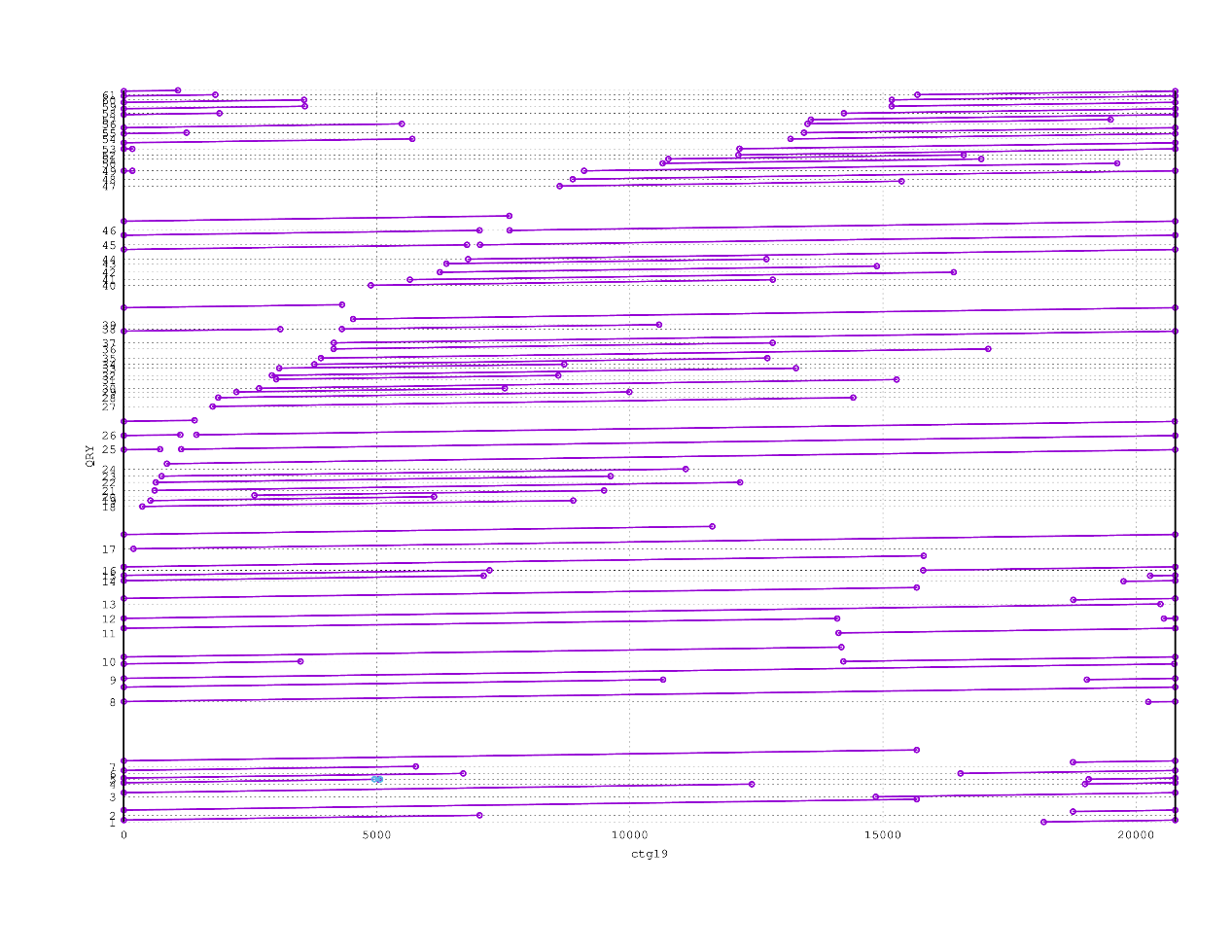


**Supplementary Figure S19. Long-read validation of the circular structure of mitochondrial contig 19.** The dot plot visualization displays the alignment of Oxford Nanopore Technologies (ONT) long reads against the assembled mitochondrial contig 19 (ctg19). The x-axis represents the reference contig sequence, and the y-axis represents individual query reads. The continuous diagonal alignments (purple lines) covering the entire length of the contig provide robust evidence for the assembly's continuity and confirm the genuine circular nature of the molecule.


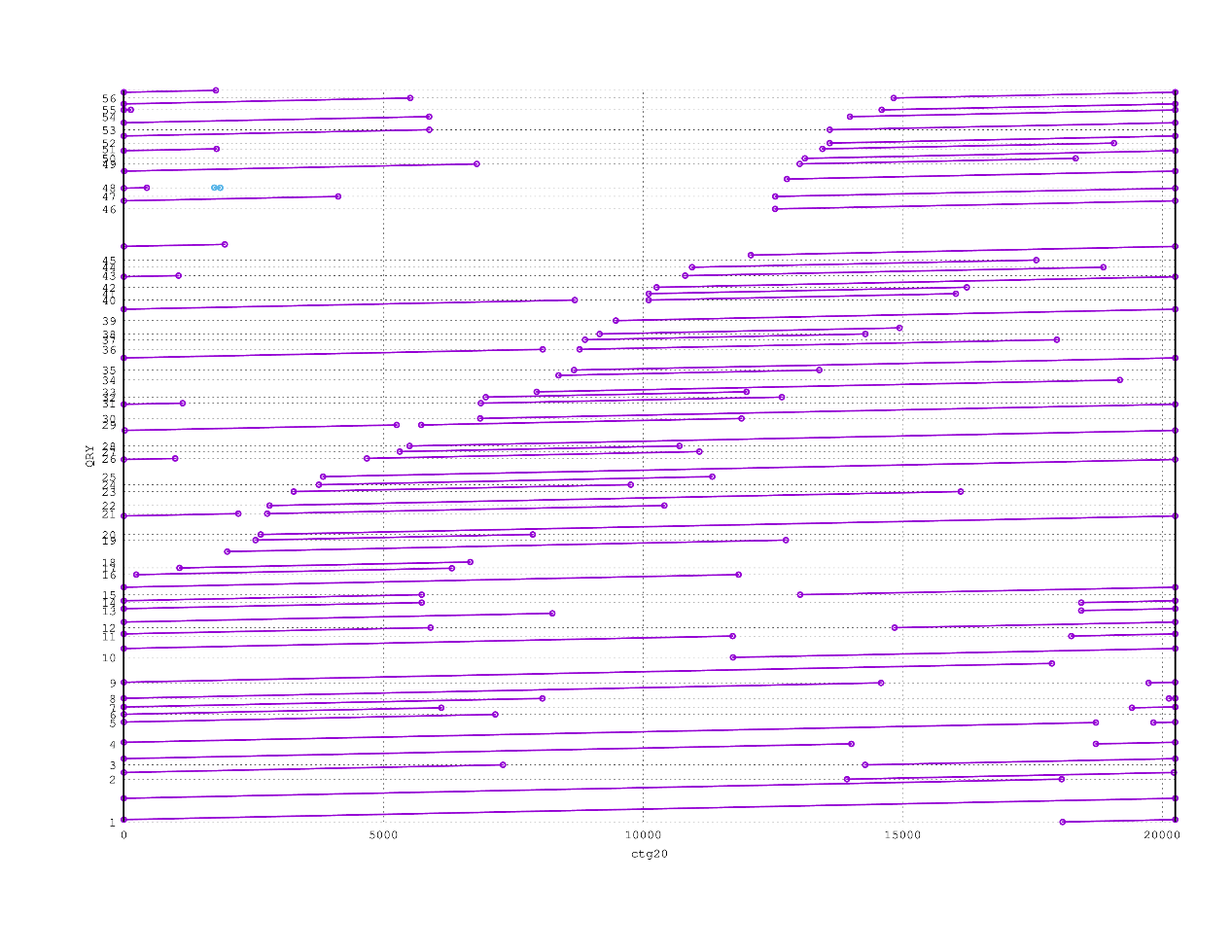


**Supplementary Figure S20. Long-read validation of the circular structure of mitochondrial contig 20.** The dot plot visualization displays the alignment of Oxford Nanopore Technologies (ONT) long reads against the assembled mitochondrial contig 20 (ctg20). The x-axis represents the reference contig sequence, and the y-axis represents individual query reads. The continuous diagonal alignments (purple lines) covering the entire length of the contig provide robust evidence for the assembly's continuity and confirm the genuine circular nature of the molecule.


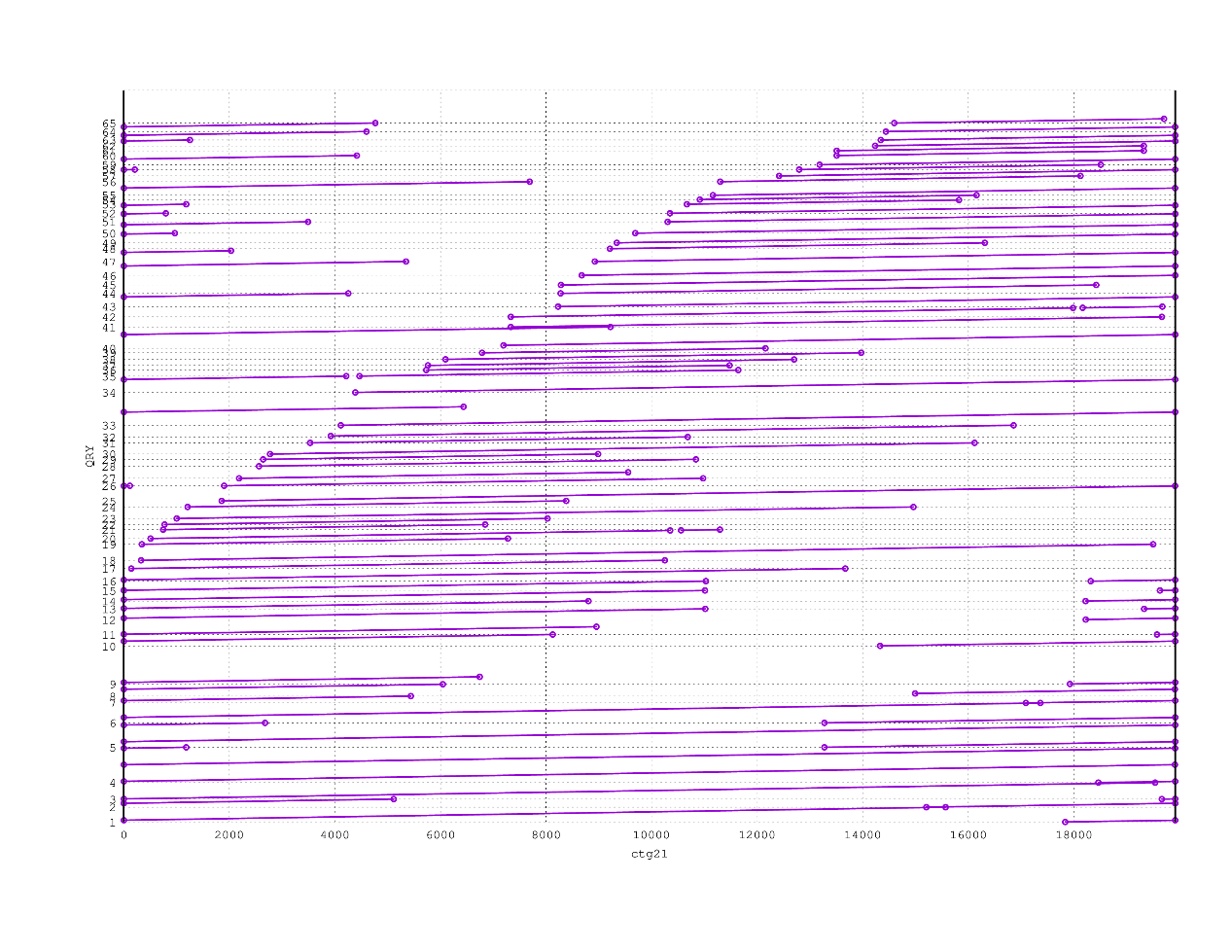


**Supplementary Figure S21. Long-read validation of the circular structure of mitochondrial contig 21.** The dot plot visualization displays the alignment of Oxford Nanopore Technologies (ONT) long reads against the assembled mitochondrial contig 21 (ctg21). The x-axis represents the reference contig sequence, and the y-axis represents individual query reads. The continuous diagonal alignments (purple lines) covering the entire length of the contig provide robust evidence for the assembly's continuity and confirm the genuine circular nature of the molecule.
